# Supplementary material for: New prolonged opioid consumption after major surgery in Sweden: a population-based retrospective cohort study
Source: BMJ Open. 2023 Apr 26;13(4):e071135. doi: 10.1136/bmjopen-2022-071135 (PMC10151846; doi:10.1136/bmjopen-2022-071135)
Supplement: Supplementary data [file bmjopen-2022-071135supp001.pdf]

Supplementary table 1: Included and excluded surgery codes.

Included surgery codes:

A – nervous system
B – endocrine system
C – eye and adjacent structures
D - ear, nose, and throat
G - pulmonary
H – mammary gland
J - gastrointestinal
K - urology
L - gynecology
N - musculoskeletal system
P - vascular
Q – dermatology

Excluded surgery codes:

F – cardiac
M – obstetric
T – minor surgical procedures
U – transluminal endoscopy
V – Surgery on aorta, peripheral vessels, and lymphatic system.
X – diagnostic procedures associated with surgery
Y – removal of organs for transplant
Z – additional codes

Minor surgical procedures include:

Neurological procedures: lumbar puncture, peripheral nerve block, sympathetic block TAW and other minor neurosurgical procedure.

Endocrine surgery: puncture of thyroid gland, needle biopsy of thyroid gland, aspiration cytology of thyroid gland, puncture of parathyroid gland, needle biopsy of parathyroid gland, needle biopsy of adrenal gland, aspiration cytology of adrenal gland, other minor procedure in endocrine surgery

Eye surgery: needle biopsy of orbit, aspiration cytology of orbit, retrobulbar therapeutic injection into orbit, peribulbar therapeutic injection into orbit, needle biopsy of eyelid, simple epilation of cilia, therapeutic injection into eyelid, removal of ptosis suture from eyelid, adjustment of ptosis suture, needle biopsy of lacrimal gland, probing of lacrimal canaliculus or of nasolacrimal duct, intubation of lacrimal canaliculus or intubation of nasolacrimal duct, therapeutic injection into extraocular muscle, subconjunctival therapeutic injection, protection of cornea and sclera with soft lens, removal of sutures from cornea, laser suture lysis in cornea or sclera, needle aspiration of aqueous from anterior chamber, needle biopsy of iris, needle biopsy of ciliary body, needle biopsy of choroid, needle biopsy of vitreous body, needle biopsy of retina, other minor procedure in eye surgery

Ear, nose and throat: biopsy of auricle or external auditory canal, needle biopsy of auricle or external auditory canal, removal of foreign body from external auditory canal, needle biopsy of tympanic membrane, needle biopsy of nose, removal of foreign body from cavity of nose, needle biopsy of septum of nose, needle biopsy of maxillary antrum, puncture and irrigation of maxillary antrum, needle biopsy of ethmoidal bone and sinus, needle biopsy of frontal sinus, needle biopsy of sphenoidal sinus, needle biopsy of larynx, other minor procedure in surgery of ear, nose and larynx

Breast surgery: needle biopsy of mamilla, needle biopsy of mammary gland, other minor procedure in surgery of mammary gland

Gastroenterological surgery: puncture of peritoneum, laparocentesis, percutaneous peritoneal irrigation, percutaneous introduction of peritoneal dialysis catheter, removal of peritoneal dialysis catheter, percutaneous local drainage of peritoneal cavity, introduction of intraperitoneal injection port, removal of intraperitoneal injection port, insertion of ballon tube for tamponade of oesophageal varices, insertion of nasogastric or naso-gastroduodenal tube, insertion of other tube into stomach or duodenum, change of gastrostomy tube, insertion of jejunal tube, insertion of jejunal tube through gastrostomy, peroral biopsy of small intestine, reduction of intestinal intussusception using barium enema, digital extraction of impacted faeces, removal of foreign body from rectum, percutaneous needle biopsy of liver, percutaneous destruction of lesion of liver, intraoperative cholangiography, laparoscopic cholangiography, percutaneous needle biopsy of pancreas, percutaneous drainage of pseudocyst or abscess of pancreas, needle biopsy of spleen, other minor procedure in gastroenterological surgery.

Urology: needle biopsy of kidney or pelvis of kidney, aspiration cytology of kidney, percutaneous puncture of kidney or pelvis of kidney, needle biopsy of bladder, percutaneous puncture of bladder, catheterisation of bladder, dilatation of urethra, needle biopsy of prostate, aspiration cytology of prostate, puncture of prostate, needle biopsy of testis, aspiration cytology of testis, puncture of testis, needle biopsy of epididymis, aspiration cytology of epididymis, puncture of epididymis, puncture of scrotum, other minor surgical procedure in urology

Gynecology: needle biopsy of ovary, needle biopsy of Fallopian tube, insertion of intrauterine contraceptive device, dilatation of vagina, removal of foreign body from vagina in childhood, transvaginal puncture of cul-de-sac, other minor surgical procedure in gynaecology

Orthopedic surgery: puncture or needle biopsy of soft tissue, simple incision of soft tissue, arthrocentesis, injection of diagnostic or therapeutic substance into joint, aspiration of bone marrow, drill biopsy of bone marrow, soft bandage, prefabricated orthosis, custom moulded splint, custom moulded cast, major custom moulded cast, other orthopedic bandage, external traction, implantation of skeletal marker

Peripheral vessels and lymphatic system: phlebotomy, open cannulation of peripheral vein, sclerotherapy of varicose veins, insertion of central venous catheter through external or internal jugular vein, insertion of central venous catheter through subclavian or brachiocephalic vein, biopsy of superficial lymph node, needle biopsy of lymph node, other minor surgical procedure on peripheral vessel or lymphatic system, biopsy of artery or vein, implantation of vascular injection port, removal of vascular injection port

Dermatology: other minor procedure on skin, biopsy of skin, needle biopsy of skin, removal of sutures of skin, adjustment of volume of tissue expander
