# Supplementary material for: New prolonged opioid consumption after major surgery in Sweden: a population-based retrospective cohort study
Source: BMJ Open. 2023 Apr 26;13(4):e071135. doi: 10.1136/bmjopen-2022-071135 (PMC10151846; doi:10.1136/bmjopen-2022-071135)
Supplement: Supplementary data [file bmjopen-2022-071135supp002.pdf]

Supplementary table 2. Risk factors for developing prolonged opioid use after surgery among patients with no opioid use 180 days before surgery. Odds ratios adjusted for sex, age and surgical procedure.

Risk factor	Crude OR (95% CI)	Adjusted OR (95% CI)[1]
**Patient factors**		
Female	1.08 (1.05-1.12)	1.16 (1.12-1.21)
Age (yr) 18-29	Ref.	Ref.
30-39	1.31 (1.19-1.44)	1.43 (1.30-1.57)
40-49	1.52 (1.40.-1.66)	1.67 (1.53-1.82)
50-59	1.90 (1.75-2.06)	1.91 (1.76-2.07)
60-69	1.96 (1.82-2.11)	1.85 (1.71-2.00)
70-79	2.19 (2.03-2.36)	1.92 (1.77-2.07)
> 80	2.80 (2.59-3.03)	2.16 (1.99-2.34)
**Preoperative data**		
Charlson comorbidity index 0p	Ref.	Ref.
1p	1.65 (1.56-1.74)	1.43 (1.35-1.51)
≥ 2p	1.42 (1.37-1.47)	1.69 (1.63-1.76)
ASA classification 1	Ref.	Ref.
2	1.83 (1.76-1.92)	1.65 (1.57-1.73)
3	2.57 (2.45-2.69)	2.25 (2.13-2.38)
4	3.07 (2.71-3.47)	2.76 (2.43-3.14)
Comorbidities		
Heart disease[†]	1.54 (1.48-1.59)	1.32 (1.27-1.37)
Lung disease[‖]	1.79 (1.69-1.91)	1.59 (1.49-1.70)
Renal disease[‡]	1.35 (1.24-1.48)	1.46 (1.33-1.60)
Diabetes mellitus	1.53 (1.46-1.61)	1.40 (1.33-1.47)
Vascular disease[↑]	1.44 (1.32-1.56)	1.44 (1.32-1.57)
Cerebrovascular disease[§]	1.50 (1.40-1.62)	1.25 (1.16-1.35)
History of psychiatric disease		
Substance abuse[¶]	1.97 (1.83-2.13)	1.99 (1.84-2.15)
Cognitive disease[ǂ]	2.15 (1.96-2.36)	1.50 (1.36-1.65)
Personality disorder, Schizophrenia[¥]	1.23 (1.12-1.35)	1.52 (1.38-1.67)
Affective disorder[£]	1.63 (1.52-1.75)	1.70 (1.59-1.83)
Anxiety disorder[®]	1.58 (1.47-1.69)	1.81 (1.68-1.95)
Preoperative Medication[♦]		
Neuroleptics	1.75 (1.63-1.89)	1.58 (1.46-1.71)
Benzodiazepines	1.84 (1.77-1.91)	1.75 (1.68-1.82)
Hypnotics & Sedatives	2.11 (2.04-2.19)	1.95 (1.89-2.02)
Antidepressants	1.90 (1.83-1.97)	1.80 (1.73-1.86)
Psychostimulants	1.70 (1.44-2.01)	2.18 (1.84-2.58)
History of psychiatric disease and/or medication**	2.10 (2.03-2.17)	1.98 (1.92-2.05)
**Perioperative factors**		
Acute surgery*	1.35 (1.30-1.39)	1.04 (1.01-1.09)
Cancer surgery	1.10 (1.05-1.14)	1.08 (1.04-1.14)

† Chronic ischemic heart disease, Angina pectoris, Hypertensive disease, Cardiac arrest, Heart failure, Valve disease, Pulmonary heart disease, Cardiomyopathy, Conduction disorders/Cardiac arrhythmias, Cardiac arrest, Diseases of arteries, arterioles and capillaries.
|| Pneumonia, COPD.
‡ Acute renal failure/unspecified renal failure, Chronic renal failure, Other renal disease.
↑Atherosclerosis, Aortic aneurysm and dissection, Other aneurysm, Other peripheral vascular diseases, Arterial embolism and thrombosis, Atheroembolism, Septic arterial embolism, Other disorders of arteries and arterioles, Diseases of capillaries, Disorders of arteries, arterioles and capillaries in diseases classified elsewhere.
§ Subarachnoid hemorrhage, Intracerebral hemorrhage, Other nontraumatic intracranial haemorrhage, Cerebral infarction, Acute cerebrovascular disease without cerebral infarction, Vascular occlusion without cerebral infarction, Other cerebrovascular diseases, Cerebrovascular disorders in diseases classified elsewhere, Sequelae of cerebrovascular disease.
¶ Mental and behavioural disorders due to psychoactive substance use.
ɨ Alzheimer's disease, Vascular dementia, Other dementia, Unspecified dementia, Non-alcoholic amnesia, Non-alcoholic delirium, mental disorders due to known physiological condition, Personality and behavioral disorders due to known physiological condition, mental disorder due to unknown somatic or organic disorder.
¥ Schizophrenia, Schizotypal disorder, Persistent delusional disorders, Acute and transient psychotic disorders, Induced delusional disorder, Schizoaffective disorders, Other nonorganic psychotic disorders, Unspecified nonorganic psychosis.
£ Manic episode, bipolar affective disorder, depressive episode, recurrent depressive disorder, persistent mood disorders, other affective mood disorders, unspecified affective mood disorders.
® Phobic anxiety disorders, other anxiety disorders, obsessive-compulsive disorder, reaction to severe stress and adjustment disorders, dissociative disorders, somatoform disorders, other neurotic disorders.
Ͱ Cognitive disease, substance abuse disorder, personality disorder/schizophrenia, affective disorder, anxiety disorder, Psychiatric medication within 5 years of surgery..
♦ Collection of prescription within five years of surgery.
* Acute surgery is defined as procedures that are admitted as acute surgeries. The level of acuteness can differ between right away, 6h and 72h depending on type of procedure.
** A diagnosis of ≥ 1 of the psychiatric diseases listed (Table 1) and/or collected a prescription of ≥ of the listed psychiatric medications (Table 1) within five years of surgery.
