# Supplementary material for: New prolonged opioid consumption after major surgery in Sweden: a population-based retrospective cohort study
Source: BMJ Open. 2023 Apr 26;13(4):e071135. doi: 10.1136/bmjopen-2022-071135 (PMC10151846; doi:10.1136/bmjopen-2022-071135)
Supplement: Supplementary data [file bmjopen-2022-071135supp003.pdf]

Supplementary table 3. *Baseline characteristics of surgical cohort at inclusion (n=216,877) and proportion of new prolonged opioid consumption.*

		New Prolonged Opioid Consumption		
	Total n = 216 877 (%)	No n = 201 796 (93)	Yes n = 15 081 (7.0)	*p*-value[2,3]
**Procedure – no (%[1])**				<0.001
Ophthalmic surgery	3 228	3 167 (98)	611 (1.9)	
Neurosurgery	14 052	13 019 (93)	1 033 (7.4)	
Endocrine surgery	7 573	7 429 (98)	144 (1.9)	
Ear, nose & throat surgery	6 839	6 666 (97)	173 2.5)	
Oral & maxillofacial surgery	8 729	8 331 (95)	398 (4.6)	
Pulmonary surgery	2 244	2 023 (90)	221 (9.9)	
Breast surgery	12 635	12 104 (96)	531 (3.5)	
Gastrointestinal surgery	43 112	40 521 (94)	2 591 (6)	
Urologic surgery	23 642	22 509 (95)	1 133 (4.8)	
Gynecologic surgery	18 994	18 412 (97)	582 (3.1)	
Orthopedic surgery	61 360	54 003 (88)	7 357 (12)	
Vascular surgery	8 450	7 965 (94)	485 (5.7)	
Dermatologic surgery	6 019	5 647 (94)	372 (6.2)	
**Year of surgery – year (%[1])**				0.52
2007-2008	42 195	39 192 (93)	5 239 (7.1)	
2009-2010	46 963	43 705 (93)	5 641 (6.9)	
2011-2012	60 213	56 067 (93)	7 174 (6.9)	
2013-2014	67 506	62 832 (93)	7 943 (6.9)	
**Region of surgery – no (%[1])**				0.009
Stockholm & Södermanland	61 142	56 935 (93)	4 207 6.9)	
Skåne	80 964	75 147 (93)	5 817 (7.2)	
Västra Götaland	37 090	34 566 (93)	2 524 (6.8)	
Västernorrland & Västerbotten	37 681	35 148 (93)	2 533 (6.7)	

[1] Percentages calculated in relation to horizontal study cohort, [2] Pearson's Chi-squared test, [3] Mann-Whitney U-test
