# Supplementary material for: New prolonged opioid consumption after major surgery in Sweden: a population-based retrospective cohort study
Source: BMJ Open. 2023 Apr 26;13(4):e071135. doi: 10.1136/bmjopen-2022-071135 (PMC10151846; doi:10.1136/bmjopen-2022-071135)
Supplement: Supplementary data [file bmjopen-2022-071135supp004.pdf]

Supplementary table 4. Sensitivity analyses of the association between history of psychiatric disease and prolonged opioid use. Restriction of the cohort; excluding patients >70 years old (75th percentile).

**Risk factor**	**OR* (95% CI)**
History of psychiatric disease	2.07 (1.99 to 2.17)

* Adjusted for sex, age, comorbidities (charlson comorbidity index) and surgical procedure.
