# Supplementary material for: New prolonged opioid consumption after major surgery in Sweden: a population-based retrospective cohort study
Source: BMJ Open. 2023 Apr 26;13(4):e071135. doi: 10.1136/bmjopen-2022-071135 (PMC10151846; doi:10.1136/bmjopen-2022-071135)
Supplement: Author's manuscript [file bmjopen-2022-071135.draft_revisions.pdf]

BMJ Open is committed to open peer review. As part of this commitment we make the peer review history of every article we publish publicly available.

When an article is published we post the peer reviewers' comments and the authors' responses online. We also post the versions of the paper that were used during peer review. These are the versions that the peer review comments apply to.

The versions of the paper that follow are the versions that were submitted during the peer review process. They are not the versions of record or the final published versions. They should not be cited or distributed as the published version of this manuscript.

BMJ Open is an open access journal and the full, final, typeset and author-corrected version of record of the manuscript is available on our site with no access controls, subscription charges or pay-per-view fees (http://bmjopen.bmj.com).

If you have any questions on BMJ Open's open peer review process please email
info.bmjopen@bmj.com

BMJ Open

New and prolonged postsurgical opioid consumption in Sweden: a population-based cohort study

Journal:	*BMJ Open*
Manuscript ID	bmjopen-2022-071135
Article Type:	Original research
Date Submitted by the Author:	20-Dec-2022

Complete List of Authors:	Lindeberg, Felix; Karolinska University Hospital, Department of Anaesthesia and Intensive Care Medicine; Karolinska Institute, Department of Physiology and Pharmacology<br>Bell, Max; Karolinska Institutet, Department of Physiology & Pharmacology; Karolinska University Hospital, Department of Physiology & Pharmacology<br>Larsson, Emma; Karolinska University Hospital, Department of Anaesthesia and Intensive Care Medicine; Karolinska Institute, Department of Physiology and Pharmacology<br>Hallqvist, Linn; Karolinska University Hospital, Department of Anaesthesia and Intensive Care Medicine; Karolinska Institute, Department of Physiology and Pharmacology

Keywords:	SURGERY, ANAESTHETICS, PAIN MANAGEMENT, Substance misuse < PSYCHIATRY, REGISTRIES, Adult surgery < SURGERY

SCHOLARONE™
Manuscripts

I, the Submitting Author has the right to grant and does grant on behalf of all authors of the Work (as defined in the below author licence), an exclusive licence and/or a non-exclusive licence for contributions from authors who are: i) UK Crown employees; ii) where BMJ has agreed a CC-BY licence shall apply, and/or iii) in accordance with the terms applicable for US Federal Government officers or employees acting as part of their official duties; on a worldwide, perpetual, irrevocable, royalty-free basis to BMJ Publishing Group Ltd ("BMJ") its licensees and where the relevant Journal is co-owned by BMJ to the co-owners of the Journal, to publish the Work in this journal and any other BMJ products and to exploit all rights, as set out in our licence.

The Submitting Author accepts and understands that any supply made under these terms is made by BMJ to the Submitting Author unless you are acting as an employee on behalf of your employer or a postgraduate student of an affiliated institution which is paying any applicable article publishing charge ("APC") for Open Access articles. Where the Submitting Author wishes to make the Work available on an Open Access basis (and intends to pay the relevant APC), the terms of reuse of such Open Access shall be governed by a Creative Commons licence – details of these licences and which Creative Commons licence will apply to this Work are set out in our licence referred to above.

Other than as permitted in any relevant BMJ Author's Self Archiving Policies, I confirm this Work has not been accepted for publication elsewhere, is not being considered for publication elsewhere and does not duplicate material already published. I confirm all authors consent to publication of this Work and authorise the granting of this licence.

For peer review only

0

New and prolonged postsurgical opioid consumption in Sweden: a population-based cohort study

Felix Lindeberg[1,2], B.Sc; Max Bell[1,2], MD, PhD; Emma Larsson[1,2], MD, PhD; Linn Hallqvist[1,2], MD, PhD.

[1] The Department of Anaesthesia and Intensive Care Medicine, Karolinska University Hospital, Stockholm, Sweden
[2] The Department of Physiology and Pharmacology, Karolinska Institute, Stockholm, Sweden.

Corresponding author:

Felix Lindeberg

Felix.lindeberg@stud.ki.se
+46767847468
Dept of Perioperative Medicine and Intensive care (PMI)
Karolinska University Hospital, Solna
Eugeniavägen 3, 171 76 STOCKHOLM
SWEDEN

For peer review only

Manuscript word count: 2954

Abstract

Objective: Given that long term opioid usage is an important problem worldwide and postsurgical pain is a common indication for opioid prescription our primary objective was to describe the frequency of new and prolonged opioid consumption after major surgery in Sweden, and, secondly, to evaluate potential associated risk factors.

Design: Cohort study including data from January 1, 2007, to December 31, 2014. Data regarding surgical procedures, baseline characteristics and outcomes was retrieved from the Orbit surgical planning system, the Swedish national patient register and the Swedish cause of death register.

Setting: Observational multicentre cohort study with data from 23 Swedish hospitals.

Participants: We included 228,584 patients aged ≥18 years, undergoing non-cardiac surgery, not exposed to opioids 90 days before, and alive 12 months after surgery.

Primary and secondary outcome measures: Primary outcome was collection of an opioid prescription between 183-365 days after surgery. Secondly, multivariable logistic regression analysis was conducted to explore potential risk factors associated with prolonged opioid use.

Results: Of the 228,584 patients identified to undergo analysis, 25,997 (11.4%) collected an opioid prescription 183-365 days after surgery. Several risk factors were identified. Higher age, a history of psychiatric disease and the combination of the two, were the most important risk factors.

Conclusion: In a large Swedish cohort of surgical patients, more than one in ten patients developed new and prolonged opioid consumption after major surgery. Our data on

For peer review only

susceptible patients could help clinicians reduce the number of prolonged opioid users by adapting their analgesic and preventative strategies.

For peer review only

Strengths and limitations of this study

- First nationwide observational cohort study investigating opioid prescription after surgery on a large Swedish population.
- Our cohort included adults of all ages being exposed to a great variety of surgeries, conducted at hospitals of all levels (university-, county-, and district). In addition, data was collected from Swedish health registers which offers robustness and great coverage.
- We are measuring dispensing of opioid prescriptions as a proxy for opioid consumption and therefore this study does not portray patient compliance to their prescription. Undeniably, we cannot account for surreptitiously opioid usage and lack data of in-hospital drug use.
- The lack of consensus in defining prolonged opioid consumption is a weakness of our study and others in the field.
- Lack of long term follow up as patients were followed for a year after surgery.

For peer review only

Introduction

The pain killing effect of opioids is a cornerstone of modern medicine but also fraught with side effects ranging from constipation and nausea to substance abuse and death [1]. More than 300 million people undergo surgery in the world every year and postsurgical pain is a common indication for opioid treatment [2,3]. Many patients are exposed to opioids for the first time due to surgery and it has been suggested that postsurgical long-term opioid use should be referred to as a surgical complication [4]. In addition, opioids are often overprescribed after surgery and many patients report taking opioids for a different indication than for which the opioids were initially prescribed for [3,5]. A previous study has indicated that patients who are prescribed opioids seven days after surgery are 50% more likely to collect an opioid prescription one year after surgery [6]. This is interesting since the proportion of patients receiving an opioid prescription at seven and 30 days after surgery differs enormously between countries [7].

Treating postsurgical pain with opioids, poses a challenge for physicians as two competing interests need to be addressed: managing acute pain and minimizing the risk of prolonged opioid use. A recent study showed that a high percentage of patients with a hip fracture, opioid naïve before surgery, were still using opioids several months after their surgical procedure [8]. Furthermore, risk factors for prolonged opioid use have been identified including type of surgery, comorbidities, and use of specific drugs ahead of surgery, e.g., antidepressants, and benzodiazepines [8-10]. Although some data exists, preoperative risk assessment might stand to gain from more data on risk factors of prolonged opioid use.

Therefore, the primary aim of the study was to describe the frequency of new and prolonged opioid consumption after major surgery in Sweden. Secondly, we sought to evaluate the

For peer review only

impact of patient factors (age, sex, medical history), surgical factors (procedure, medical treatment) and healthcare system related factors (regional differences and time trends). We hypothesized that differences associated with both patient- and surgical characteristics exist.

For peer review only

Materials and Methods

Study Design & Participants

This was an observational multicentre cohort study using data from 23 Swedish hospitals at university-, county-, and district level, representing approximately 40 percent of the Swedish perioperative care. Patients aged ≥ 18 years, undergoing surgery from January 1, 2007, to December 31, 2014, were included. Exclusion criteria were: collection of an opioid prescription within 90 days ahead of surgery, not being alive 12 months after surgery, undergoing cardiac, obstetric, ambulatory, minor (see supplementary table x), or multiple surgeries as well as missing a valid surgery code in Orbit or a corresponding surgery code in National Patient Register (NPR). Additionally, patients identified from hospitals with a high proportion of missing American Society of Anaesthesiologists (ASA) physical status classification were also excluded (Figure 1). The study protocol was approved by the Regional Ethics Committee of Stockholm, Sweden (2014/1306-31/3).

Data sources

Orbit - surgical planning system

Orbit is a surgical planning system used by Swedish hospitals and provides information about patient characteristics, ASA physical status classification, date, type and duration of surgery and anaesthesia, all linked to the Swedish personal identification number. The system was used to identify the study cohort.

Health Registers

For peer review only

Data extracted from Orbit were linked to the (NPR) using the unique Swedish personal identification number (PIN) assigned at birth or immigration [11]. The NPR provides information of all hospital admissions since the year of 1987 and outpatient care since 2001 [12]. The Swedish prescribed drug register contains information about all dispensed *and collected* drug prescriptions in Sweden since July 2005 [13]. In the Swedish cause of death register virtually all (> 99%) deaths are registered since 1952 based on the current ICD-classification [14].

For peer review only

Data collection

Data was retrieved from the above-mentioned registers. Data collection included information about patient characteristics and medical history such as age, sex, ASA physical status classification, hospital diagnoses and collected drug prescriptions. This was made possible by backtracking the NPR and the Swedish prescribed drug register for five years before surgery and one year after surgery. Data from the Swedish cause of death register was collected for one year after surgery. Perioperative characteristics included date, type, and duration of surgery and was extracted from Orbit. Surgical procedures were clustered into twelve subtypes based on surgical codes used in the Nordic countries (NOMESCO). Subtypes included gastrointestinal; endocrine; ophthalmic; ear, nose, and throat; thoracic; neuro; breast; urologic; gynaecologic; orthopaedic; vascular; and dermatologic surgery.

Primary endpoint

The primary endpoint was new and prolonged opioid use, defined as collection of an opioid prescription within 183-365 days after surgery in a previously opioid naïve patient. Being

opioid naïve was defined as *not collecting* an opioid prescription within 90 days prior to surgery.

Secondary Aims

Secondary aims were to evaluate the impact of patient-, surgical-, and healthcare system related risk factors for prolonged opioid prescription in the opioid naïve patient. Patient risk factors included age, sex, as well as somatic and psychiatric comorbidities, surgical risk factors include type of surgery and medical treatment, whereas healthcare related risk factors are differences associated with four different regions in Sweden and the year of surgery.

Statistical Analysis

The study cohort was divided into two groups based on meeting the primary outcome or not. Categorical variables within each of these groups are presented as percentages of the total population and continuous data as medians with interquartile ranges (IQR) that represent 25-75th percentiles. Continuous variables were analysed using the Mann-Whitney U test and categorical variables were compared with the Chi-squared test generating p-values to look for statistical differences between the groups. P-values < 0.05 were considered significant and statistical tests were two-sided. Logistic regression analysis was conducted to investigate the relationships between potential pre- and perioperative, patient-, and surgery-related risk factors and the associated effect on our primary endpoint. Variables with univariate significance, and considered clinically relevant, were explored in the multivariate analysis to explore our secondary aims. Hence, crude and adjusted odds ratios, presented with 95% confidence intervals, were calculated to see how the risk estimates changed when adjusting

For peer review only

for potential confounders. Adjustments for age, sex, type of surgery and year of surgery was performed in the final model. Patients that died within one year of surgery were excluded due to competing risk. A sensitivity analysis including these patients were conducted which had little effect on the results. Additional sensitivity analysis and tests for interaction were conducted; with restriction of the cohort to patients >70 years of age, excluding patients in the 75th percentile. and analyses of interaction between the covariates age >70 years and history of psychiatric disease. The combination age <70 years and no history of psychiatric was considered to represent the lowest risk and used as reference. Data was analysed by STATA version 14.2 (Stata Corp., College Station, TX, USA).

Patient and Public Involvement

It was not appropriate or possible to involve patients or the public in the design, or conduct, or reporting, or dissemination plans of our research.

For peer review only

Results

Patient characteristics

228,584 patients were identified to undergo analysis (Figure 1). 48,469 patients were excluded due to collection of an opioid prescription within 90 days of surgery and 13,253 patients were excluded because they died within 12 months of surgery (Figure 1). The population consisted of 125,693 (55%) women and had a median (IQR) age of 62 (45, 73) years old (Table 1). 22,691 (10%) of patients had a history of psychiatric disease and 85,050 (37%) had collected a prescription for psychiatric medication up to five years before surgery (Table 1). 161,553 (71%) of the conducted surgeries were elective and orthopaedic surgery was the most common procedure (Table 1). 87,499 (38%) patients were discharged from the hospital with an opioid prescription (Table 1).

For peer review only

Table 1. Baseline characteristics of surgical cohort at inclusion (n=228 584) and proportion of new postsurgical prolonged opioid use

		Dispensing of Opioid Prescription 183-365 days after surgery		
	Total n = 228 584 (%)	No n = 202 587 (88.6)	Yes n = 25 997 (11.4)	*p*-value[2,3]
Age – median years (IQR)	62 (45, 73)	61 (44, 72)	65 (51, 76)	<0.001
Women – no. (%[1])	125 693 (55)	110 652 (55)	15 041 (58)	<0.001
**Age range – no. (%[1])**				<0.001
Age 18-29	23 630 (10)	22 104 (11)	1 526 (5.9)	
Age 30-39	19 296 (8.4)	17 609 (8.7)	1 687 (6.5)	
Age 40-49	26 962 (12)	24 282 (12)	2 680 (10)	
Age 50-59	33 824 (15)	30 013 (15)	3 811 (15)	
Age 60-69	52 306 (23)	46 330 (23)	5 976 (23)	
Age 70-79	43 966 (19)	38 248 (19)	5 718 (22)	
Age >80	28 600 (13)	24 001 (12)	4 599 (18)	
**Preoperative data – no. (%[1])**				
ASA Classification				<0.001
ASA1	76 667 (34)	71 248 (35)	5 419 (21)	
ASA2	102 198 (45)	89 915 (44)	12 283 (47)	
ASA3	46 972 (21)	39 212 (19)	7 760 (30)	
ASA4	2 747 (1.2)	2 212 (1.1)	535 (2.1)	
Charlson comorbidity index				<0.001
0	131 722 (58)	119 395 (59)	12 327 (47)	
1	19 792 (9)	16 873 (8)	2 919 (11)	
>2	77 070 (34)	66 319 (33)	10 751 (41)	
History of psychiatric disease	22 691 (10)	10 101 (9)	3 590 (14)	<0.001
Heart disease	55 754 (24)	47 202 (23)	8 552 (33)	<0.001
Lung disease	11 356 (5.0)	9 234 (4.6)	2 122 (8.2)	<0.001
Renal disease	6 615 (2.9)	5 485 (2.7)	1 130 (4.3)	<0.001
Diabetes Mellitus	15 709 (6.9)	13 007 (6.4)	2 702 (10)	<0.001
Peripheral vascular disease	4 756 (3.3)	6 245 (3.1)	1 211 (4.7)	<0.001
Cerebrovascular disease	8 894 (3.9)	7 421 (3.7)	1 473 (5.7)	<0.001
Infectious disease	2 762 (1.2)	2 246 (1.1)	516 (2.0)	<0.001
Cognitive disease	4 149 (1.8)	3 327 (1.6)	822 (3.2)	<0.001
Substance abuse disease	6 638 (2.9)	5 413 (2.7)	1 225 (4.7)	<0.001
Personality disorder, schizophrenia	6 128 (2.7)	5 278 (2.6)	850 (3.3)	<0.001
Affective disorders	9 119 (4.0)	7 549 (3.7)	1 570 (6.0)	<0.001
Anxiety disorders	9 513 (4.2)	7 924 (3.9)	1 589 (6.1)	<0.001
**Preoperative medication – no (%[1])**				
Psychiatric medication	85 050 (37)	71 124 (35)	13 926 (54)	<0.001

Neuroleptics	7 532 (3.3)	6 187 (3.1)	1 345 (5.2)	<0,001
Benzodiazepines	38 778 (17)	32 045 (16)	6 733 (26)	<0,001
Hypnotics & Sedatives	52 440 (23)	43 037 (21)	9 403 (36)	<0,001
Antidepressants	44 444 (19)	36 776 (18)	7 668 (29)	<0,001
Psychostimulants	1 536 (0.7)	1 264 (0.6)	272 (1.0)	<0,001
Anti-dementia drugs	2 279 (1.0)	1 832 (0.9)	447 (1.7)	<0,001
**Type of surgery – no. (%[1])**				< 0.001
Acute surgery	67 051 (29)	58 724 (29)	8 327 (32)	
Elective surgery	161 533 (71)	143 863 (71)	17 670 (68)	
Cancer surgery	45 762 (20)	39 908 (20)	5 854 (23)	<0.001
Time of surgery – min. (IQR)	82 (50, 129)	82 (50, 128)	84 (50, 134)	<0,001
**Procedure – no (%[1])**				<0.001
Ophthalmic surgery	3 425 (1.4)	3 184 (1.5)	241 (0.9)	
Neurosurgery	15 051 (6.6)	13 072 (6.5)	1 979 (7.6)	
Endocrine surgery	7 875 (3.4)	7 317 (3.6)	558 (2.1)	
Ear, nose & throat surgery	7 067 (3.1)	6 549 (3.2)	518 (2.0)	
Oral & maxillofacial surgery	9003 (3.9)	8 322 (4.1)	681 (2.6)	
Pulmonary surgery	2 364 (1.0)	2 056 (1.0)	308 (1.2)	
Breast surgery	13 086 (5.7)	11 855 (5.9)	1 231 (4.7)	
Gastrointestinal surgery	45 204 (20)	40 307 (20)	4 897 (19)	
Urologic surgery	24 623 (11)	22 345 (11)	2 278 (8.8)	
Gynaecologic surgery	19 689 (8.6)	18 064 (8.9)	1 625 (6.3)	
Orthopaedic surgery	66 123 (29)	56 245 (28)	9 878 (38)	
Vascular surgery	8 871 (3.9)	7 782 (3.8)	1 089 (4.2)	
Dermatologic surgery	6 275 (2.7)	5 553 (2.7)	722 (2.8)	
**Opioid prescription – no (%[1])**				
Opioid at discharge	87 499 (38)	74 320 (37)	13 179 (51)	<0.001
Opioid at 31-90 days	20 103 (8.8)	12 780 (6.3)	7 323 (28)	<0.001
Opioid at 91-182 days	18 387 (8.0)	9 479 (4.7)	8 908 (34)	<0.001
**Opioid type at discharge – no. (%[1])**				
Opiates, e.g., Morphine	61 319 (27)	51 782 (26)	9 537 (37)	<0.001
Fentanyl & Ketogan	1 284 (0.6)	982 (0.5)	302 (1.2)	<0.001
Methadone	8 131 (3.6)	7 064 (3.5)	1 067 (4.1)	<0.001
Buprenorphine, e.g., Suboxone	170 (0.1)	99 (0.1)	71 (0.3)	<0.001
Opioids with antispasmodics	352 (0.2)	296 (0.1)	56 (0.2)	0.007
Other opioids, e.g., Tramadol	20 013 (8.8)	16 957 (8.4)	3 056 (12)	<0.001
**Year of surgery – year (%[1])**				<0.001
2007-2008	47 421 (20)	41 772 (20)	5 649 (20)	
2009-2010	52 540 (22)	46 438 (22)	6 102 (22)	
2011-2012	67 394 (28)	59 645 (28)	7 749 (28)	
2013-2014	74 482 (31)	66 182 (31)	8 300 (30)	

[1] Percentages calculated in relation to vertical study cohort, [2] Pearson's Chi-squared test, [3] Mann-Whitney U-test

Primary endpoint

A total number of 25,997 patients (11.4%), opioid naïve before surgery, collected an opioid prescription within 183-365 days of surgery (Table 1).

Multivariable analysis of risk factors associated with prolonged opioid use after surgery

Estimated adjusted odds ratios (aOR) are presented in Figure 2. Odds ratios were adjusted for sex, age, comorbidities, surgical procedure, and year of surgery. Postoperative prolonged opioid use was associated with female sex (aOR: 1.11. 95% CI: 1.08-1.14). In terms of the age, the risk increased with the consolidated age categories spanning across approximately 10 years where the aOR for the oldest group was 1.61 (95% CI: 1.51-1.72). Compared to a Charlson comorbidity index (CCI) of 0p, the risk increased with a higher index. CCI of 1p had an aOR of 1.35 (95% CI: 1.29-1.41) and CCI ≥ 2 had an aOR of 1.44 (95% CI: 1.39-1.49). Having a history of psychiatric disease and/or psychiatric medication use yielded an aOR of 1.91 (95% CI: 1.86-1.96). Estimated aORs for ASA class, individual comorbidities, preoperative medications, type of surgery and postoperative opioid consumption are presented in a supplementary figure.

Regarding perioperative factors acute surgery had an aOR at 1.09 (95% CI: 1.06-1.13) compared to elective surgery and cancer surgery had an aOR of 1.25 (95% CI: 1.20-1.49).

For peer review only

Sensitivity analysis

Several sensitivity analyses were conducted. The results remained similar when including patients that died within 12 months of surgery in the analysis as well as exploring different regions and years separately. As higher age and history of psychiatric disease seemed to be associated with elevated risk of prolonged opioid consumption, we performed sensitivity analyses, using the adjusted model with exclusion of patients >70 years, in the upper 4th quartile. (n=67 132). This yielded a higher risk estimate regarding history of psychiatric disease (aOR: 2.02. 95% CI: 1.96-2.10), indicating that the impact of a psychiatric disease on risk of prolonged opioid consumption might be higher in younger patients (Table 2). In a further interaction analysis, we created 4 risk groups based on one or a combination of the two risk factors: age <70 years (75th percentile) and a history of psychiatric disease (Table 2). Age >70 entailed the lowest risk (OR 1.51), a history of psychiatric disease a higher risk (OR 2.16) and the combination an even higher risk (OR 2.76) (Table 2).

Table 2. Interaction analyses of age and history of psychiatric disease.
OR of prolonged opioid use after surgery in the presence of a one or a combination of the two risk factors: age >70 years (Q4) and a history of psychiatric disease.

**Risk**	**OR (95% CI)**	***P***
Risk1[a]	1.51 (1.45 to 1.57)	<0.001
Risk2[b]	2.16 (2.10 to 2.23)	<0.001
Risk3[c]	2.76 (2.66 to 2.86)	<0.001
Risk0[f] (ref)	omitted	

[a]risk1: age >70 years and no history of psychiatric disease
[b]risk2: age <70 years and a history of psychiatric disease
[c]risk3: age >70 years and a history of psychiatric disease
[f]risk0: age <70 years and no history of psychiatric disease

For peer review only

Discussion

In this large nation-wide retrospective perioperative cohort study, 11.4% developed new and prolonged opioid use. Factors associated with a higher risk of prolonged opioid use were female sex, higher age, more comorbidities, history of psychiatric disease, acute surgery, and cancer surgery. Additionally, a combination of higher age and a history of psychiatric disease increased the risk further.

Strengths

This is the first retrospective observational cohort study investigating opioid prescription after surgery on a large Swedish population. Additionally, to our knowledge, the number of included patients outnumbers many other similar studies on an international level. Utilizing the Orbit surgical planning system this study was able to include patients from different regions all around Sweden covering approximately 40% of Swedish perioperative care. This high external validity means that we believe the results to be generalizable in other Swedish settings. Furthermore, the robustness and great coverage of Swedish health registers is also a strength of this study [12]. Our cohort included adults of all ages being exposed to a great variety of surgeries. Despite not having information about socioeconomic status our data should represent the actual healthcare burden of the Swedish population. The public healthcare system with services being provided according to patient need limits the selection bias that healthcare systems with various private insurances may generate.

Limitations

For peer review only

Some limitations of our study merit emphasis. First, our study was observational and therefore does not prove that our identified risk factors portray a causal relationship. Moreover, since a large part of our data was extracted from various Swedish registers misclassification bias could also have played a part in the reporting of data. However, the potential misclassification bias should be non-differential in nature and hence not push the results one direction or the other. The relatively old data is a potentially limiting factor. But, to our knowledge, there has been no major changes in Swedish postoperative pain management or legislation affecting opioid prescription patterns in recent years. This notion is also supported by the absence of differences over time in our study which makes us believe more recent data would yield similar results. Another important aspect is the fact that we are measuring dispensing of opioid prescriptions as a proxy for opioid consumption. This study does not portray patient compliance to their prescription. However, regardless of actual opioid consumption dispensing of prescriptions are problematic in and of themselves as it increases the number of opioids circulating in communities. Undeniably, we cannot account for surreptitiously opioid usage either and lack data of in-hospital drug use, something that could influence postoperative opioid usage. Additionally, as we excluded patients dispensing opioid prescriptions before surgery, we cannot extrapolate our results to patients that are opioid dependent before surgery. A weakness of this study and others in the field is the lack of consensus in defining prolonged opioid consumption. This complicates comparing results in between studies. Furthermore, there is also no consensus for how long patients should be opioid free before surgery.

Postoperative prolonged opioid use

Compared to others, this study found a higher occurrence of new and prolonged opioid use after surgery [4,6,9,15]. Two cohort studies of patients aged 66 or older conducted in the U.S. and

For peer review only

Canada found new postoperative prolonged opioid use at about 6% and 3%, respectively [4,9]. Sun *et al.,* studied patients between the age of 18-64 and found prolonged opioid use at around 1% [15]. The discrepancy in occurrence of postoperative prolonged opioid use between these studies can at least partially be explained by differences in definitions of prolonged opioid use and how long a patient must be opioid naïve for. Sun *et al.,* used the most conservative definition of prolonged use where patients needed to fill 10 or more prescriptions or more than 120 days' supply of an opioid in the first year after surgery [15]. While this definition captures the patients with a high postsurgical opioid consumption it may underestimate the risk for patients with a lower, yet problematic, use. Although some patients will need opioids to manage postoperative acute pain, this period should be limited. Previous studies have shown that half of patients using opioids for longer than 90 days persist in their usage for many years after initiation [16,17]. This highlights the importance of restrictiveness in prescribing opioids. Two of these studies only included privately insured patients [4,15]. This introduces a bias in only selecting patients with the ability to afford a private insurance, potentially yielding a healthier population that is not generalizable to the actual population of the country. Another aspect that could lead to differences in results between these studies is the lack of consensus regarding how to handle patients that died before the end of follow-up. We excluded these patients before analysis to avoid competing risk. In the Canadian cohort patients that were unable to meet the primary outcome were also excluded [9] whereas the three other studies do not state this [4,6,15].

Patient related risk factors for postoperative opioid use

The risk of prolonged opioid use was higher for women. This is not a consistent finding across the literature as other studies have found male sex [15] to be a greater risk factor, or no significant gender difference [4,8,9]. Regarding age, the oldest patients had the highest aOR in

For peer review only

this study. A similar trend was observed regarding Charlson comorbidity index, where a higher score generated a higher aOR. Higher age as a risk factor is not a consistent finding. Other studies have found both the oldest and the youngest population to be at higher risk, but also no significant difference in terms of age [4,8,9,15]. History of psychiatric disease and/or medication led to almost a two-fold risk increase. Excluding the oldest population further increased the aOR for history of psychiatric disease indicating that the impact of psychiatric disease as a risk factor for new postoperative prolonged opioid use is stronger in younger patients. In line with this, other studies have found an association between preoperative benzodiazepine or antidepressant use, and prolonged postoperative opioid consumption [10,18]. When we created four risk groups based on one or a combination of the two risk factors; age > 70 years (75th percentile) and a history of psychiatric disease it appeared that the combination of the two yielded the highest risk. Our results, alongside aforementioned studies, indicate that clinicians must be aware of this relationship when prescribing postoperative opioids, especially when a patient fits into both these risk groups. In selected cases, other modes of analgesia may be considered as first line of pain treatment postoperatively for these high-risk-patients. If opioids are still regarded necessary, prescriptions with shorter duration and fewer pills should be applied since over-prescription have been identified as a problem [5].

Surgery related risk factors for postoperative opioid use

Acute surgery had a higher occurrence of postoperative opioid use compared to elective surgery. Being exposed to cancer surgery yielded an aOR of 1.25 (1.20-1.49). A patient going through cancer surgery is a potential confounder in our study as treating cancer patients with opioids often is unavoidable. Partly, we tried to account for this by excluding patients prescribed opioids up to 90 days before surgery. Moreover, since individuals dying up to a

For peer review only

year after surgery were excluded, we were hopefully able to account for unsuccessful surgical removal of the tumour and deterioration of advanced stages of malignancies.

Conclusion

In this large national observational cohort study, more than one in ten patients developed new postoperative prolonged opioid use. We identified several potential risk factors; with higher age, history of psychiatric disease and the combination of the two, being the most important. Data on these susceptible patients could help clinicians reduce the number of prolonged opioid users by adapting their analgesic and preventative strategies. Clinicians need be aware of the risks, have a plan for tapering, and realize that every opioid prescription has the potential of turning a non-user into a long-term user.

For peer review only

Data availability statement

Data may be obtained from a third party and are not publicly available. Data from Swedish health registers are available to all researchers upon making an application.

Ethics statements

Patient consent for publication

Not applicable.

Ethics approval

The study protocol was approved by the Regional Ethics Committee of Stockholm, Sweden (2014/1306-31/3).

Author Contributions

FL, MB and LH were responsible for the study concept and design. LH did the data collection and data cleaning. FL and LH did the data analysis. FL drafted the manuscript. MB, EL and LH did critical review of the manuscript. All the authors interpreted the data and approved the final manuscript as submitted and agree to be accountable for all aspects of the work.

Funding Statement

This research received no specific grant from any funding agency in the public, commercial or not-for-profit sectors.

For peer review only

Competing Interests Statement

None of the authors have any competing interests to declare.

For peer review only

References

1. Nafziger AN, Barkin RL. Opioid Therapy in Acute and Chronic Pain. *J Clin Pharmacol*. Sep 2018;58(9):1111-1122. doi:10.1002/jcph.1276

2. Weiser TG, Haynes AB, Molina G, et al. Estimate of the global volume of surgery in 2012: an assessment supporting improved health outcomes. *Lancet*. Apr 27 2015;385 Suppl 2:S11. doi:10.1016/s0140-6736(15)60806-6

3. Callinan CE, Neuman MD, Lacy KE, Gabison C, Ashburn MA. The Initiation of Chronic Opioids: A Survey of Chronic Pain Patients. *J Pain*. Apr 2017;18(4):360-365. doi:10.1016/j.jpain.2016.11.001

4. Brummett CM, Waljee JF, Goesling J, et al. New Persistent Opioid Use After Minor and Major Surgical Procedures in US Adults. *JAMA Surg*. Jun 21 2017;152(6):e170504. doi:10.1001/jamasurg.2017.0504

5. Hartwell MJ, Selley RS, Terry MA, Tjong VK. Can We Eliminate Opioid Medications for Postoperative Pain Control? A Prospective, Surgeon-Blinded, Randomized Controlled Trial in Knee Arthroscopic Surgery. *Am J Sports Med*. Sep 2020;48(11):2711-2717. doi:10.1177/0363546520941861

6. Alam A, Gomes T, Zheng H, Mamdani MM, Juurlink DN, Bell CM. Long-term analgesic use after low-risk surgery: a retrospective cohort study. *Arch Intern Med*. Mar 12 2012;172(5):425-30. doi:10.1001/archinternmed.2011.1827

7. Ladha KS, Neuman MD, Broms G, et al. Opioid Prescribing After Surgery in the United States, Canada, and Sweden. *JAMA Netw Open*. Sep 4 2019;2(9):e1910734. doi:10.1001/jamanetworkopen.2019.10734

8. Edwards NM, Varnum C, Overgaard S, Nikolajsen L, Christiansen CF, Pedersen AB. Risk factors for new chronic opioid use after hip fracture surgery: a Danish nationwide cohort study from 2005 to 2016 using the Danish multidisciplinary hip fracture registry. *BMJ Open*. Mar 8 2021;11(3):e039238. doi:10.1136/bmjopen-2020-039238

9. Clarke H, Soneji N, Ko DT, Yun L, Wijeysundera DN. Rates and risk factors for prolonged opioid use after major surgery: population based cohort study. *BMJ*. Feb 11 2014;348:g1251. doi:10.1136/bmj.g1251

10. Rajamäki TJ, Moilanen T, Puolakka PA, Hietaharju A, Jämsen E. Is the Preoperative Use of Antidepressants and Benzodiazepines Associated with Opioid and Other Analgesic Use After Hip and Knee Arthroplasty? *Clin Orthop Relat Res*. Oct 1 2021;479(10):2268-2280. doi:10.1097/corr.0000000000001793

11. Ludvigsson JF, Otterblad-Olausson P, Pettersson BU, Ekbom A. The Swedish personal identity number: possibilities and pitfalls in healthcare and medical research. *Eur J Epidemiol*. 2009;24(11):659-67. doi:10.1007/s10654-009-9350-y

12. Ludvigsson JF, Andersson E, Ekbom A, et al. External review and validation of the Swedish national inpatient register. *BMC Public Health*. Jun 9 2011;11:450. doi:10.1186/1471-2458-11-450

13. Socialstyrelsen. Läkemedelsregistret. Socialstyrelsen. Accessed 2022-05-24, 2022. https://www.socialstyrelsen.se/statistik-och-data/register/lakemedelsregistret/

14. Socialstyrelsen. Dödsorsaksregistret. https://www.socialstyrelsen.se/statistik-och-data/register/dodsorsaksregistret/

15. Sun EC, Darnall BD, Baker LC, Mackey S. Incidence of and Risk Factors for Chronic Opioid Use Among Opioid-Naive Patients in the Postoperative Period. *Jama Internal Medicine*. Sep 2016;176(9):1286-1293. doi:10.1001/jamainternmed.2016.3298

16. Von Korff M, Saunders K, Thomas Ray G, et al. De facto long-term opioid therapy for noncancer pain. *Clin J Pain*. Jul-Aug 2008;24(6):521-7. doi:10.1097/AJP.0b013e318169d03b

For peer review only

17. Martin BC, Fan MY, Edlund MJ, Devries A, Braden JB, Sullivan MD. Long-term chronic opioid therapy discontinuation rates from the TROUP study. *J Gen Intern Med*. Dec 2011;26(12):1450-7. doi:10.1007/s11606-011-1771-0

18. Rishel CA, Zhang Y, Sun EC. Association Between Preoperative Benzodiazepine Use and Postoperative Opioid Use and Health Care Costs. *JAMA Netw Open*. Oct 1 2020;3(10):e2018761. doi:10.1001/jamanetworkopen.2020.18761

For peer review only

Figure legends

Figure 1. Participant flowchart

Figure 1. Risk factors for developing new and prolonged opioid use after surgery. Odds ratios are presented as mutually adjusted for age, sex, comorbidities, surgical procedure, and year of surgery.

eFigure1. Risk factors for developing new and prolonged opioid use after surgery. Odds ratios are presented as adjusted for age and sex.

For peer review only

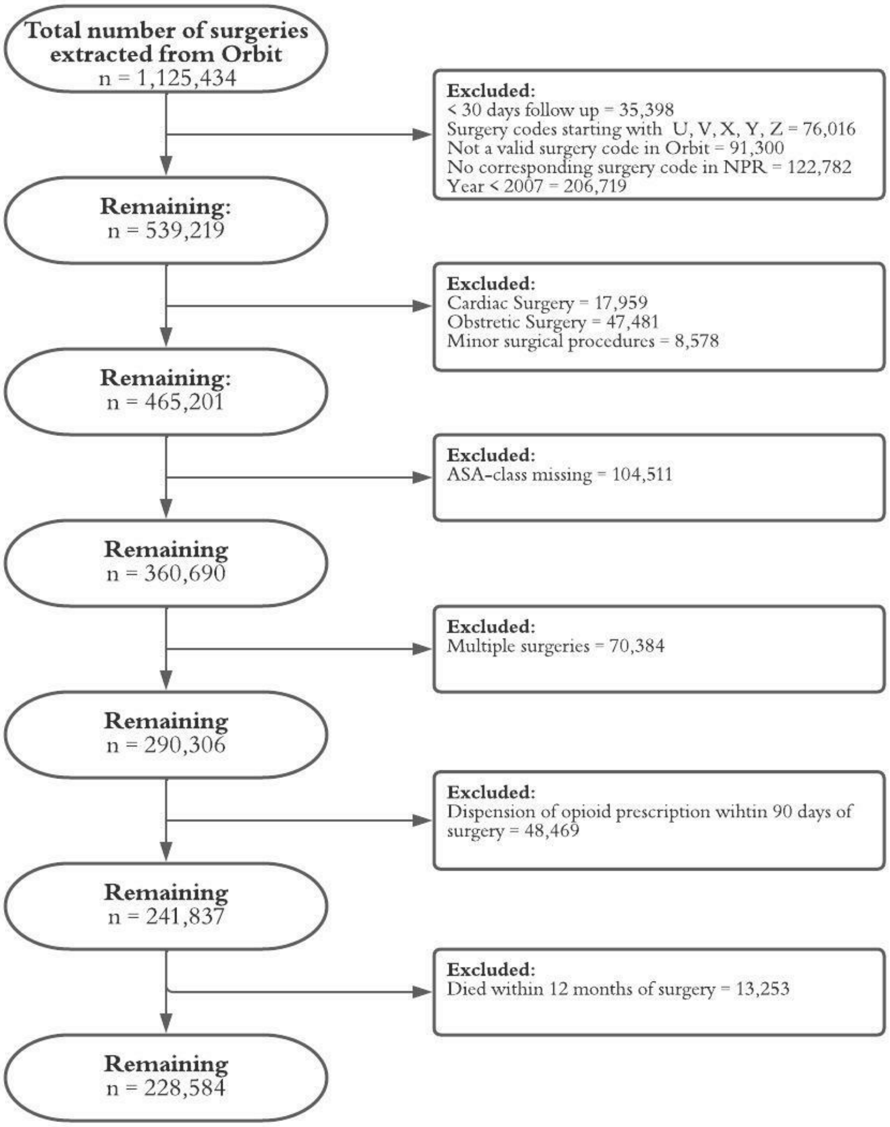

Participant flowchart

142x177mm (160 x 160 DPI)

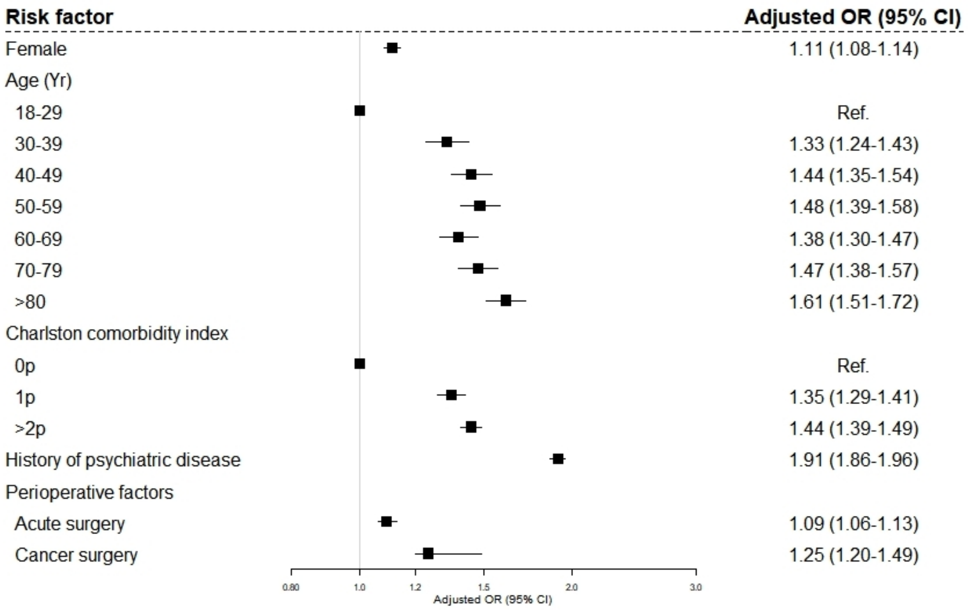

Risk factors for developing new and prolonged opioid use after surgery. Odds ratios are presented as mutually adjusted for age, sex, comorbidities, surgical procedure, and year of surgery.

228x158mm (96 x 96 DPI)

0

**eTable 1.** Baseline characteristics of surgical cohort at inclusion (n=228 584) and proportion of new postsurgical prolonged opioid use.				
		Dispensing of Opioid Prescription 183-365 days after surgery		
	Total n = 228 584 (%)	No n = 202 587 (88.6)	Yes n = 25 997 (11.4)	*p*-value[2,3]
**Procedure – no (%[1])**				<0.001
Ophthalmic surgery	3 353 (100)	3 120 (93)	233 (6.9)	
Neurosurgery	15 051 (100)	13 072 (87)	1 979 (13)	
Endocrine surgery	7 875 (100)	7 317 (93)	558 (7.0)	
Ear, nose & throat surgery	7 067 (100)	6 549 (93)	518 (7.0)	
Oral & maxillofacial surgery	9003 (100)	8 322 (92)	681 (8.0)	
Pulmonary surgery	2 364 (100)	2 056 (87)	308 (13)	
Breast surgery	13 086 (100)	11 855 (91)	1 231 (9.0)	
Gastrointestinal surgery	45 204 (100)	40 307 (89)	4 897 (11)	
Urologic surgery	24 623 (100)	22 345 (91)	2 278 (9.0)	
Gynaecologic surgery	19 689 (100)	18 064 (92)	1 625 (8.3)	
Orthopaedic surgery	66 123 (100)	56 245 (85)	9 878 (15)	
Vascular surgery	8 871 (100)	7 782 (88)	1 089 (12)	
Dermatologic surgery	6 275 (100)	5 553 (88)	722 (12)	
**Year of surgery – year (%[1])**				<0.001
2007-2008	46 672 (100)	39 433 (88)	5 239 (12)	
2009-2010	49 510 (100)	43 869 (89)	5 641 (11)	
2011-2012	63 428 (100)	56 254 (89)	7 174 (11)	
2013-2014	70 974 (100)	63 031 (89)	7 943 (11)	
**Region of surgery – no (%[1])**				0.13
Västernorrland & Västerbotten	39 635 (100)	35 303 (89)	4 332 (11)	
Stockholm & Södermanland	64 438 (100)	56 856 (88)	7 582 (12)	
Skåne	85 686 (100)	75 881 (89)	9 805 (11)	
Västra Götaland	38 825 (100)	34 547 (89)	4 278 (11)	

[1] Percentages calculated in relation to horizontal study cohort, [2] Pearson's Chi-squared test, [3] Mann-Whitney U-test

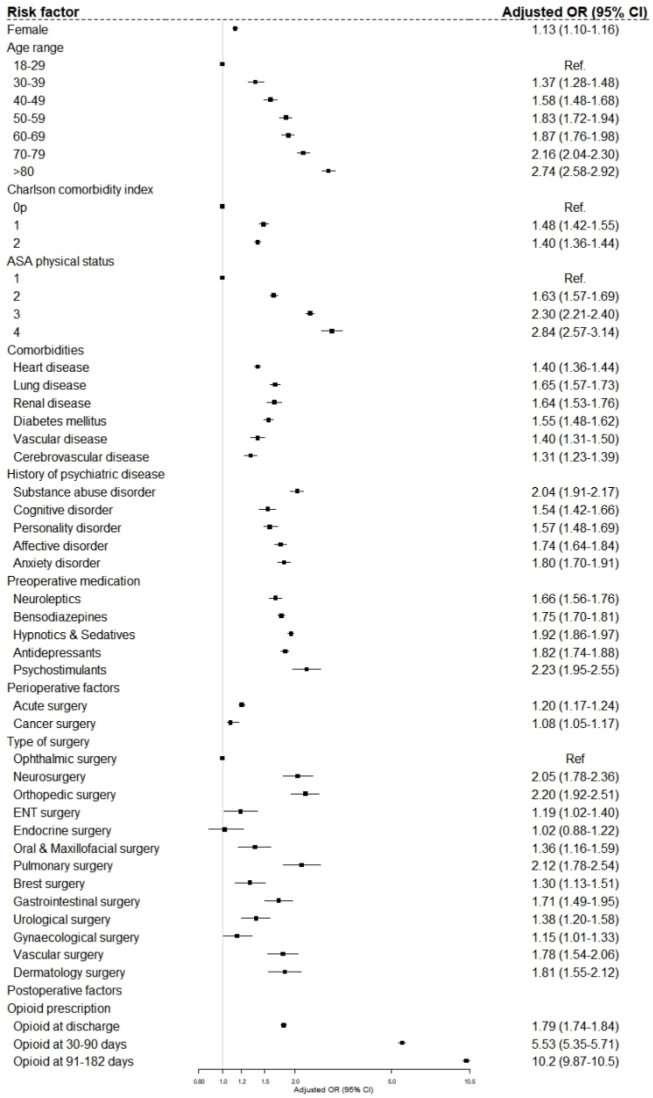

Risk factors for developing new and prolonged opioid use after surgery. Odds ratios are presented as adjusted for age and sex.

228x407mm (96 x 96 DPI)

STROBE Statement—Checklist of items that should be included in reports of ***cohort studies***

	Item No	Recommendation	Page No
**Title and abstract**	1	(*a*) Indicate the study's design with a commonly used term in the title or the abstract	1
		(*b*) Provide in the abstract an informative and balanced summary of what was done and what was found	1
**Introduction**			
Background/rationale	2	Explain the scientific background and rationale for the investigation being reported	3
Objectives	3	State specific objectives, including any prespecified hypotheses	3-4
**Methods**			
Study design	4	Present key elements of study design early in the paper	5-6
Setting	5	Describe the setting, locations, and relevant dates, including periods of recruitment, exposure, follow-up, and data collection	5
Participants	6	(*a*) Give the eligibility criteria, and the sources and methods of selection of participants. Describe methods of follow-up	5
		(*b*) For matched studies, give matching criteria and number of exposed and unexposed	N/A
Variables	7	Clearly define all outcomes, exposures, predictors, potential confounders, and effect modifiers. Give diagnostic criteria, if applicable	6-7
Data sources/ measurement	8*	For each variable of interest, give sources of data and details of methods of assessment (measurement). Describe comparability of assessment methods if there is more than one group	5-6
Bias	9	Describe any efforts to address potential sources of bias	8
Study size	10	Explain how the study size was arrived at	Figure 1
Quantitative variables	11	Explain how quantitative variables were handled in the analyses. If applicable, describe which groupings were chosen and why	7
Statistical methods	12	(*a*) Describe all statistical methods, including those used to control for confounding	7
		(*b*) Describe any methods used to examine subgroups and interactions	7
		(*c*) Explain how missing data were addressed	7
		(*d*) If applicable, explain how loss to follow-up was addressed	N/A
		(*e*) Describe any sensitivity analyses	10
**Results**			
Participants	13*	(a) Report numbers of individuals at each stage of study—eg numbers potentially eligible, examined for eligibility, confirmed eligible, included in the study, completing follow-up, and analysed	Figure 1
		(b) Give reasons for non-participation at each stage	5 & Figure 1
		(c) Consider use of a flow diagram	Figure 1
Descriptive data	14*	(a) Give characteristics of study participants (eg demographic, clinical, social) and information on exposures and potential confounders	5, 9 & Table 1
		(b) Indicate number of participants with missing data for each variable of interest	Table 1

For peer review only - http://bmjopen.bmj.com/site/about/guidelines.xhtml

		(c) Summarise follow-up time (eg, average and total amount)	6
Outcome data	15*	Report numbers of outcome events or summary measures over time	9 & Table 1
Main results	16	(*a*) Give unadjusted estimates and, if applicable, confounder-adjusted estimates and their precision (eg, 95% confidence interval). Make clear which confounders were adjusted for and why they were included<br>(*b*) Report category boundaries when continuous variables were categorized<br>(*c*) If relevant, consider translating estimates of relative risk into absolute risk for a meaningful time period	9, 10 & Table 1<br>7-8
Other analyses	17	Report other analyses done—eg analyses of subgroups and interactions, and sensitivity analyses	10
**Discussion**			
Key results	18	Summarise key results with reference to study objectives	9, 10
Limitations	19	Discuss limitations of the study, taking into account sources of potential bias or imprecision. Discuss both direction and magnitude of any potential bias	14, 15
Interpretation	20	Give a cautious overall interpretation of results considering objectives, limitations, multiplicity of analyses, results from similar studies, and other relevant evidence	11-13
Generalisability	21	Discuss the generalisability (external validity) of the study results	13-14
**Other information**			
Funding	22	Give the source of funding and the role of the funders for the present study and, if applicable, for the original study on which the present article is based	16

*Give information separately for exposed and unexposed groups.

Note: An Explanation and Elaboration article discusses each checklist item and gives methodological background and published examples of transparent reporting. The STROBE checklist is best used in conjunction with this article (freely available on the Web sites of PLoS Medicine at http://www.plosmedicine.org/, Annals of Internal Medicine at http://www.annals.org/, and Epidemiology at http://www.epidem.com/). Information on the STROBE Initiative is available at http://www.strobe-statement.org.

For peer review only

BMJ Open

New prolonged opioid consumption after major surgery in Sweden: a population-based retrospective cohort study

Journal:	*BMJ Open*
Manuscript ID	bmjopen-2022-071135.R1
Article Type:	Original research
Date Submitted by the Author:	31-Mar-2023

Complete List of Authors:	Lindeberg, Felix; Karolinska University Hospital, Department of Anaesthesia and Intensive Care Medicine; Karolinska Institute, Department of Physiology and Pharmacology<br>Bell, Max; Karolinska Institutet, Department of Physiology & Pharmacology; Karolinska University Hospital, Department of Physiology & Pharmacology<br>Larsson, Emma; Karolinska University Hospital, Department of Anaesthesia and Intensive Care Medicine; Karolinska Institute, Department of Physiology and Pharmacology<br>Hallqvist, Linn; Karolinska University Hospital, Department of Anaesthesia and Intensive Care Medicine; Karolinska Institute, Department of Physiology and Pharmacology

**Primary Subject Heading**:	Surgery
Secondary Subject Heading:	Medical management, Anaesthesia
Keywords:	SURGERY, ANAESTHETICS, PAIN MANAGEMENT, Substance misuse < PSYCHIATRY, REGISTRIES, Adult surgery < SURGERY

SCHOLARONE™
Manuscripts

I, the Submitting Author has the right to grant and does grant on behalf of all authors of the Work (as defined in the below author licence), an exclusive licence and/or a non-exclusive licence for contributions from authors who are: i) UK Crown employees; ii) where BMJ has agreed a CC-BY licence shall apply, and/or iii) in accordance with the terms applicable for US Federal Government officers or employees acting as part of their official duties; on a worldwide, perpetual, irrevocable, royalty-free basis to BMJ Publishing Group Ltd ("BMJ") its licensees and where the relevant Journal is co-owned by BMJ to the co-owners of the Journal, to publish the Work in this journal and any other BMJ products and to exploit all rights, as set out in our licence.

The Submitting Author accepts and understands that any supply made under these terms is made by BMJ to the Submitting Author unless you are acting as an employee on behalf of your employer or a postgraduate student of an affiliated institution which is paying any applicable article publishing charge ("APC") for Open Access articles. Where the Submitting Author wishes to make the Work available on an Open Access basis (and intends to pay the relevant APC), the terms of reuse of such Open Access shall be governed by a Creative Commons licence – details of these licences and which Creative Commons licence will apply to this Work are set out in our licence referred to above.

Other than as permitted in any relevant BMJ Author's Self Archiving Policies, I confirm this Work has not been accepted for publication elsewhere, is not being considered for publication elsewhere and does not duplicate material already published. I confirm all authors consent to publication of this Work and authorise the granting of this licence.

For peer review only

0

New prolonged opioid consumption after major surgery in Sweden: a population-based retrospective cohort study

Felix Lindeberg[1,2], B.Sc; Max Bell[1,2], MD, PhD; Emma Larsson[1,2], MD, PhD; Linn Hallqvist[1,2], MD, PhD.

[1] The Department of Anaesthesia and Intensive Care Medicine, Karolinska University Hospital, Stockholm, Sweden
[2] The Department of Physiology and Pharmacology, Karolinska Institute, Stockholm, Sweden.

Corresponding author:

Felix Lindeberg

Felix.lindeberg@stud.ki.se
+46767847468
Dept of Perioperative Medicine and Intensive care (PMI)
Karolinska University Hospital, Solna
Eugeniavägen 3, 171 76 STOCKHOLM
SWEDEN

Manuscript word count: 2898

For peer review only

Abstract

Objective: Given that long term opioid usage is an important problem worldwide and postsurgical pain is a common indication for opioid prescription our primary objective was to describe the frequency of new prolonged opioid consumption after major surgery in Sweden, and, secondly, to evaluate potential associated risk factors.

Design: Cohort study including data from January 1, 2007, to December 31, 2014. Data regarding surgical procedures, baseline characteristics and outcomes was retrieved from the Orbit surgical planning system, the Swedish national patient register and the Swedish cause of death register.

Setting: Observational multicentre cohort study with data from 23 Swedish hospitals.

Participants: We included 216,877 patients aged ≥18 years, undergoing non-cardiac surgery, not exposed to opioids 180 days before, and alive 12 months after surgery.

Primary and secondary outcome measures: The primary endpoint was collection of at least three opioid prescriptions during the first postoperative year; within 90 days, day 91-180 and 181-365 after surgery in a previously opioid naïve patient. Secondly, multivariable logistic regression analysis was conducted to explore potential risk factors associated with prolonged opioid use.

Results: Of the 216,877 patients identified to undergo analysis, 15,081 (7.0%) developed new prolonged opioid consumption. Several risk factors were identified. Having a history of psychiatric disease was identified as the strongest risk factor (aOR: 1.94. 95% CI: 1.87-2.00).

Conclusion: In a large Swedish cohort of surgical patients, 7% developed new prolonged opioid consumption after major surgery. Our data on susceptible patients could help clinicians reduce the number of prolonged opioid users by adapting their analgesic and preventative strategies.

For peer review only

Strengths and limitations of this study

- First nationwide observational cohort study investigating opioid prescription after major surgery on a large Swedish population.
- The study cohort include adults of all ages, exposed to various major surgeries, and data is collected from Swedish health registers which offers robustness and great coverage.
- The lack of consensus in defining prolonged opioid consumption is a weakness of our study and others in the field.
- This study does not portray patient compliance to their prescription as dispensing of opioid prescriptions is a proxy for opioid consumption.
- Lack of long-term follow up, after the first postoperative year.

For peer review only

Introduction

The analgesic effect of opioids is a cornerstone of modern medicine but also fraught with side effects ranging from constipation and nausea to substance abuse and death [1]. More than 300 million people undergo surgery in the world every year and postsurgical pain is a common indication for opioid treatment [2, 3]. Many patients are exposed to opioids for the first time due to surgery and it has been suggested that postsurgical long-term opioid use should be referred to as a surgical complication [4]. In addition, opioids are often overprescribed after surgery and many patients report taking opioids for a different indication than for which the opioids were initially prescribed for [3, 5]. A previous study has indicated that patients who are prescribed opioids seven days after surgery are 50% more likely to collect an opioid prescription one year after surgery [6]. This is interesting since the proportion of patients receiving an opioid prescription at seven and 30 days after surgery differs enormously between countries [7].

Treating postsurgical pain with opioids, poses a challenge for physicians as two competing interests need to be addressed: managing acute pain and minimizing the risk of prolonged opioid use. A recent study showed that a high percentage of patients with a hip fracture, opioid naïve before surgery, were still using opioids several months after their surgical procedure [8]. Furthermore, risk factors for prolonged opioid use have been identified including type of surgery, comorbidities, and use of specific drugs ahead of surgery, e.g., antidepressants, and benzodiazepines [8-10]. Although some data exists, preoperative risk assessment might stand to gain from more data on risk factors of prolonged opioid use.

Therefore, the primary aim of the study was to describe the frequency of new prolonged opioid consumption after major surgery in Sweden. Data from a Swedish cohort would add insight to the field as most of similar studies have been conducted on North American

For peer review only

cohorts. Secondly, we sought to evaluate the impact of patient factors (age, sex, medical history), surgical factors (procedure, medical treatment) and healthcare system related factors (regional differences and time trends). Based on earlier studies from other countries we hypothesized that differences associated with both patient- and surgical characteristics exist.

For peer review only

Materials and Methods

Study Design & Participants

This was an observational multicentre cohort study using data from 23 Swedish hospitals at university-, county-, and district level, covering approximately 40 percent of the Swedish perioperative care. Included hospitals are located in different legislative regions and dispersed geographically throughout the country which makes the data generalizable to the whole Swedish population. Patients aged ≥ 18 years, undergoing surgery from January 1, 2007, to December 31, 2014, were included. Exclusion criteria were: collection of an opioid prescription within 180 days ahead of surgery, not being alive 12 months after surgery, undergoing cardiac, obstetric, ambulatory, minor (supplementary table 1), or multiple surgeries as well as missing a valid surgery code in Orbit or a corresponding surgery code in National Patient Register (NPR). Additionally, patients identified from hospitals with a high proportion of missing American Society of Anaesthesiologists (ASA) physical status classification were also excluded (Figure 1). The study protocol was approved by the Regional Ethics Committee of Stockholm, Sweden (2014/1306-31/3).

Data sources

Orbit - surgical planning system

Orbit is a surgical planning system used by Swedish hospitals and provides information about patient characteristics, ASA physical status classification, date, type and duration of surgery and anaesthesia, all linked to the Swedish personal identification number. Surgery cannot be performed without admitting a patient to orbit. The system was used to identify the study cohort.

For peer review only

Health Registers

Data extracted from Orbit were linked to the (NPR) using the unique Swedish personal identification number (PIN) assigned at birth or immigration [11]. The NPR provides information of all hospital admissions since the year of 1987 and outpatient care since 2001 [12]. The Swedish prescribed drug register contains information about all dispensed *and collected* drug prescriptions in Sweden since July 2005 [13]. In the Swedish cause of death register virtually all (> 99%) deaths are registered since 1952 based on the current ICD-classification [14].

Data collection

Data was retrieved from the above-mentioned registers. Data collection included information about patient characteristics and medical history such as age, sex, ASA physical status classification, hospital diagnoses and collected drug prescriptions. This was made possible by backtracking the NPR and the Swedish prescribed drug register for five years before surgery and one year after surgery. Data from the Swedish cause of death register was collected for one year after surgery. Perioperative characteristics included date, type, and duration of surgery and was extracted from Orbit. Surgical procedures were clustered into twelve subtypes based on surgical codes used in the Nordic countries (NOMESCO). Subtypes included gastrointestinal; endocrine; ophthalmic; ear, nose, and throat; thoracic; neuro; breast; urologic; gynaecologic; orthopaedic; vascular; and dermatologic surgery.

Primary endpoint

For peer review only

The primary endpoint was new prolonged opioid use, defined as collection of at least three opioid prescriptions during the first postoperative year; within 90 days, day 91-180 and 181-365 after surgery in a previously opioid naïve patient. Being opioid naïve was defined as not collecting an opioid prescription within 180 days prior to surgery.

Secondary Aims

Secondary aims were to evaluate the impact of patient-, surgical-, and healthcare system related risk factors for prolonged opioid prescription in the opioid naïve patient. Patient risk factors included age, sex, as well as somatic and psychiatric comorbidities, surgical risk factors include type of surgery and medical treatment, whereas healthcare related risk factors are differences associated with four different regions in Sweden and the year of surgery.

Statistical Analysis

The data was analysed during the autumn of 2022. The study cohort was divided into two groups based on meeting the primary outcome or not. Categorical variables within each of these groups are presented as percentages of the total population and continuous data as medians with interquartile ranges (IQR) that represent 25-75th percentiles. Continuous variables were analysed using the Mann-Whitney U test and categorical variables were compared with the Chi-squared test generating p-values to look for statistical differences between the groups. P-values < 0.05 were considered significant and statistical tests were two-sided. Logistic regression analysis was conducted to investigate the relationships between potential pre- and perioperative, patient-, and surgery-related risk factors and the associated effect on our primary endpoint. Variables with univariate significance, and considered

For peer review only

clinically relevant, were explored in the multivariate analysis to explore our secondary aims, results showed in a supplementary table 2. Hence, crude and adjusted odds ratios, presented with 95% confidence intervals, were calculated to see how the risk estimates changed when adjusting for potential confounders. Adjustments for age, sex, comorbidities, year of surgery and type of surgery (including cancer surgery) was performed in the final model. Patients that died within one year of surgery were excluded due to competing risk. A sensitivity analysis including these patients were conducted which had little effect on the results. A history of psychiatric disease was significantly associated with elevated risk of prolonged opioid consumption, and we hypothesized that the impact of this risk factor might be different depending on age. Therefore, a restriction analysis was performed, using the adjusted model with exclusion of patients >70 years, in the upper 4th quartile (n=67 132). Data was analysed by STATA version 14.2 (Stata Corp., College Station, TX, USA).

Patient and Public Involvement

It was not possible to involve patients or the public in the design, or conduct, or reporting, or dissemination plans of our research.

For peer review only

Results

Patient characteristics

216,877 patients were identified to undergo analysis (Figure 1). 61,004 patients were excluded due to collection of an opioid prescription within 180 days of surgery and 12,425 patients were excluded because they died within 12 months of surgery (Figure 1). The population consisted of 118,698 (55%) women and had a median (IQR) age of 62 (45, 72) years old (Table 1). 21,169 (10%) of patients had a history of psychiatric disease and 78,743 (36%) had collected a prescription for psychiatric medication up to five years before surgery (Table 1). 152,204 (70%) of the conducted surgeries were elective and orthopaedic surgery was the most common procedure (Table 1).

For peer review only

Table 1. Baseline characteristics of surgical cohort at inclusion (n=216 877) and proportion of new prolonged opioid consumption

		New Prolonged Opioid Consumption		
	Total n = 216 877 (%)	No n = 201 796 (93)	Yes n = 15 081 (7.0)	*p*-value[2,3]
Age – median years (IQR)	62 (45, 72)	61 (44, 72)	66 (52, 76)	<0.001
Women – no. (%[1])	118 698 (55)	110 173 (55)	8 525 (57)	<0.001
**Age range – no. (%[1])**				<0.001
Age 18-29	22 996 (11)	22 118 (11)	878 (5.8)	
Age 30-39	18 509 (8.5)	17 593 (8.7)	916 (6.1)	
Age 40-49	25 634 (12)	24 171 (12)	1 463 (9.7)	
Age 50-59	31 973 (15)	29 732 (15)	2 241 (15)	
Age 60-69	49 472 (23)	45 894 (23)	3 578 (24)	
Age 70-79	41 394 (19)	38 989 (19)	3 314 (22)	
Age >80	26 899 (12)	24 208 (12)	2 691 (18)	
**Preoperative data – no. (%[1])**				
ASA Classification				<0.001
ASA1	74 067 (34)	70 947 (35)	3 120 (21)	
ASA2	96 381 (44)	89 173 (44)	7 208 (48)	
ASA3	43 853 (20)	39 406 (20)	4 447 (29)	
ASA4	2 576 (1.2)	2 270 (1.1)	306 (2.0)	
Charlson comorbidity index				<0.001
0	125 868 (58)	118 447 (59)	7 421 (49)	
1	18 408 (8.5)	16 684 (8.3)	1 724 (11)	
>2	72 601 (33)	66 319 (33)	10 751 (41)	
Heart disease[†]	51 693 (24)	46 904 (23)	4 796 (32)	<0.001
Lung disease[‖]	10 457 (4.8)	9 259 (4.6)	1 198 (7.9)	<0.001
Renal disease[‡]	6 079 (2.8)	5 525 (2.7)	554 (3.7)	<0.001
Diabetes Mellitus	19 630 (9.0)	17 694 (8.8)	1 936 (13)	<0.001
Peripheral vascular disease[↑]	6 837 (3.2)	6 182 (3.1)	655 (4.3)	<0.001
Cerebrovascular disease[§]	8 323 (3.8)	7 496 (3.7)	827 (5.5)	<0.001
Cognitive disease[ǂ]	3 908 (1.8)	3 375 (1.7)	533 (3.5)	<0.001
Substance abuse disease[¶]	6 191 (2.9)	5 413 (2.7)	788 (5.2)	<0.001
Personality disorder, schizophrenia[¥]	5 795 (2.7)	5 310 (2.6)	485 (2.6)	<0.001
Affective disorders[£]	8 435 (3.9)	7 536 (3.7)	899 (6.0)	<0.001
Anxiety disorders[®]	8 774 (4.1)	7 867 (3.9)	907 (6.0)	<0.001
History of psychiatric disease[□]	21 169 (10)	19 056 (9.4)	2 113 (14)	<0.001
**Preoperative medication – no (%[1])[♦]**				
Psychiatric medication[Ψ]	78 743 (36)	70 692 (35)	8 051 (53)	<0.001
Neuroleptics	7 007 (3.2)	6 211 (3.1)	796 (5.3)	<0,001
Benzodiazepines	35 721 (16)	31 853 (16)	3 868 (26)	<0,001
Hypnotics & Sedatives	48 117 (22)	42 660 (21)	5 457 (36)	<0,001

For peer review only - http://bmjopen.bmj.com/site/about/guidelines.xhtml

Antidepressants	41 008 (19)	36 549 (18)	4 459 (30)	<0,001
Psychostimulants	1 431 (0.7)	1 270 (0.6)	161 (1.1)	<0,001
Anti-dementia drugs	2 171 (1.0)	1 867 (0.9)	304 (2.0)	<0,001
**Type of surgery – no. (%[1])**				< 0.001
Acute surgery *	64 673 (30)	59 259 (29)	5 414 (36)	
Elective surgery	152 204 (70)	142 537 (71)	9 667 (64)	
Cancer surgery	43 881 (20)	40 613 (20)	3 268 (22)	<0.001
Time of surgery – min. (IQR)	82 (50, 129)	82 (50, 128)	88 (55, 138)	<0,001
**Procedure – no (%[1])**				<0.001
Ophthalmic surgery	3 228 (1.5)	3 167 (1.6)	611 (0.4)	
Neurosurgery	14 052 (6.5)	13 019 (6.5)	1 033 (6.9)	
Endocrine surgery	7 573 (3.5)	7 429 (3.7)	144 (1.0)	
Ear, nose & throat surgery	6 839 (3.2)	6 666 (3.3)	173 (1.2)	
Oral & maxillofacial surgery	8 729 (4.0)	8 331 (4.1)	398 (2.6)	
Pulmonary surgery	2 244 (1.0)	2 023 (1.0)	221 (1.2)	
Breast surgery	12 635 (5.8)	12 104 (6.0)	531 (3.5)	
Gastrointestinal surgery	43 112 (20)	40 521 (20)	2 591 (17)	
Urologic surgery	23 642 (11)	22 509 (11)	1 133 (7.5)	
Gynaecologic surgery	18 94 (8.8)	18 412 (9.1)	582 (3.9)	
Orthopaedic surgery	61 360 (28)	54 003 (27)	7 357 (49)	
Vascular surgery	8 450 (3.9)	7 965 (4.0)	485 (3.2)	
Dermatologic surgery	6 019 (2.8)	5 647 (2.8)	372 (2.5)	
**Opioid type at discharge – no. (%[1])**				
Opiates, e.g., Morphine	57 015 (26)	48 882 (24)	8 133 (54)	<0.001
Fentanyl & Ketogan	1 179 (0.5)	922 (0.5)	257 (1.7)	<0.001
Methadone	7 675 (3.5)	6 747 (3.3)	928 (6.2)	<0.001
Buprenorphine, e.g., Suboxone	139 (0.1)	86 (0.04)	53 (0.4)	<0.001
Opioids w/ antispasmodics, e.g. Tarqiniq	317 (0.2)	269 (0.1)	48 (0.3)	<0.001
Other opioids, e.g., Tramadol	18 430 (8.5)	15 964 (7.9)	2 466 (16)	<0.001
**Year of surgery – year (%[1])**				<0.001
2007-2008	42 195 (20)	39 192 (19)	3 003 (20)	
2009-2010	46 963 (22)	43 705 (22)	3 258 (22)	
2011-2012	60 213 (28)	56 067 (28)	4 146 (27)	
2013-2014	67 506 (31)	62 832 (31)	4 674 (31)	

[1] Percentages calculated in relation to vertical study cohort, [2] Pearson's Chi-squared test, [3] Mann-Whitney U-test

† Chronic ischemic heart disease, Angina pectoris, Hypertensive disease, Cardiac arrest, Heart failure, Valve disease, Pulmonary heart disease, Cardiomyopathy, Conduction disorders/Cardiac arrhythmias, Cardiac arrest, Diseases of arteries, arterioles and capillaries.

|| Pneumonia, COPD.

‡ Acute renal failure/unspecified renal failure, Chronic renal failure, Other renal disease.

↑Atherosclerosis, Aortic aneurysm and dissection, Other aneurysm, Other peripheral vascular diseases, Arterial embolism and thrombosis, Atheroembolism, Septic arterial embolism, Other disorders of arteries and arterioles, Diseases of capillaries, Disorders of arteries, arterioles and capillaries in diseases classified elsewhere.

§ Subarachnoid hemorrhage, Intracerebral hemorrhage, Other nontraumatic intracranial haemorrhage, Cerebral infarction, Acute cerebrovascular disease without cerebral infarction, Vascular occlusion without cerebral infarction, Other cerebrovascular diseases, Cerebrovascular disorders in diseases classified elsewhere, Sequelae of cerebrovascular disease.

ǂ Alzheimer's disease, Vascular dementia, Other dementia, Unspecified dementia, Non-alcoholic amnesia, Non-alcoholic delirium, mental disorders due to known physiological condition, Personality and behavioral disorders due to known physiological condition, mental disorder due to unknown somatic or organic disorder.
¶ Mental and behavioural disorders due to psychoactive substance use.
¥ Schizophrenia, Schizotypal disorder, Persistent delusional disorders, Acute and transient psychotic disorders, Induced delusional disorder, Schizoaffective disorders, Other nonorganic psychotic disorders, Unspecified nonorganic psychosis.
£ Manic episode, bipolar affective disorder, depressive episode, recurrent depressive disorder, persistent mood disorders, other affective mood disorders, unspecified affective mood disorders.
® Phobic anxiety disorders, other anxiety disorders, obsessive-compulsive disorder, reaction to severe stress and adjustment disorders, dissociative disorders, somatoform disorders, other neurotic disorders.
□ Cognitive disease, substance abuse disorder, personality disorder/schizophrenia, affective disorder, anxiety disorder, Psychiatric medication within 5 years of surgery.
Ψ Collection of ≥ 1 of the listed psychiatric medications (Neuroleptics, Benzodiazepines, Hypnotics & Sedatives, Antidepressants, Antipsychotics, Anti-dementia drugs) within five years of surgery.
♦ Collection of prescription within five years of surgery.
* Acute surgery is defined as procedures that are admitted as acute surgeries. The level of acuteness can differ between right away, 6h and 72h depending on type of procedure.

For peer review only

Primary endpoint

A total number of 15,081 patients (7.0 %), opioid naïve before surgery, collected at least three opioid prescriptions during the first postoperative year; within 90 days, day 91-180 and 181-365 after surgery (Table 1).

Multivariable analysis of risk factors associated with prolonged opioid use after surgery

Estimated adjusted odds ratios (aOR) are presented in Figure 2. Odds ratios were adjusted for sex, age, comorbidities, year of surgery, and type of surgery. Postoperative prolonged opioid use was associated with female sex (aOR: 1.08. 95% CI: 1.05-1.12). In terms of the age, there was no striking trend, and the highest risk was found for patients between 50-59 years of age (aOR: 1.52. 95% CI: 1.40-1.65). Compared to a Charlson comorbidity index (CCI) of 0p, the risk increased with a higher index. CCI of 1p had an aOR of 1.31 (95% CI: 1.24-1.39) and CCI ≥ 2 had an aOR of 1.36 (95% CI: 1.30-1.43). Having a history of psychiatric disease and/or psychiatric medication use yielded an aOR of 1.94 (95% CI: 1.87-2.00). Estimated

aORs for ASA class, individual comorbidities and preoperative medications are presented in supplementary table 2.

Regarding perioperative factors acute surgery had an aOR at 1.12 (95% CI: 1.04-1.17) compared to elective surgery and cancer surgery had an aOR of 1.06 (95% CI: 1.02-1.14).

Sensitivity analysis

Several sensitivity analyses were conducted. The results remained similar when including patients that died within 12 months of surgery in the analysis. No significant time trends or regional differences were identified (supplementary table 3). As history of psychiatric disease was associated with elevated risk of prolonged opioid consumption, a restriction analysis was performed, using the adjusted model with exclusion of patients >70 years, in the upper 4th quartile (n=67 132). This yielded a higher risk estimate regarding history of psychiatric disease (aOR: 2.07. 95% CI: 1.99-2.17), indicating that the impact of a psychiatric disease on risk of prolonged opioid consumption might be higher in younger patients, detailed in supplementary table 4.

For peer review only

Discussion

In this large nation-wide retrospective perioperative cohort study, 7 % developed new prolonged opioid use. Factors associated with a higher risk of prolonged opioid use were female sex, more comorbidities, and acute surgery. Additionally, having a history of psychiatric disease was identified as the strongest risk factor.

Strengths

This is the first retrospective observational cohort study investigating opioid prescription after surgery on a large Swedish population. Additionally, to our knowledge, the number of included patients outnumbers many other similar studies on an international level. Utilizing the Orbit surgical planning system this study was able to include patients from different regions all around Sweden covering approximately 40% of Swedish perioperative care. Given inclusion of hospitals from different regions and levels, we believe the results to be representative of the remaining ~ 60% of Swedish care and hence, generalizable in other Swedish settings. Furthermore, the robustness and great coverage of Swedish health registers is also a strength of this study [12]. Our cohort included adults of all ages being exposed to a great variety of surgeries. Despite not having information about socioeconomic status our data should represent the actual healthcare burden of the Swedish population. The public healthcare system with services being provided according to patient need limits the selection bias that healthcare systems with various private insurances may generate.

Limitations

For peer review only

The most important weakness of this study and others in the field is the lack of consensus in defining prolonged opioid consumption. This complicates comparing results in between studies. Furthermore, there is also no consensus for how long patients should be opioid free before surgery.

Some other limitations of our study merit emphasis. First, our study was observational and therefore does not prove that our identified risk factors portray a causal relationship. Moreover, since a large part of our data was extracted from various Swedish registers misclassification bias could also have played a part in the reporting of data. However, the potential misclassification bias should be non-differential in nature and hence not push the results one direction or the other. The relatively old data is a potentially limiting factor. But, to our knowledge, there has been no major changes in Swedish postoperative pain management or legislation affecting opioid prescription patterns in recent years. This notion is also supported by the absence of differences over time in our study which makes us believe more recent data would yield similar results. Another important aspect is the fact that we are measuring dispensing of opioid prescriptions as a proxy for opioid consumption. This study does not portray patient compliance to their prescription. However, regardless of actual opioid consumption dispensing of prescriptions are problematic in and of themselves as it increases the number of opioids circulating in communities. Undeniably, we cannot account for surreptitiously opioid usage either and lack data of in-hospital drug use, something that could influence postoperative opioid usage.

Postoperative prolonged opioid use

Compared to others, this study found a higher occurrence of new prolonged opioid use after surgery [4, 6, 9, 15]. Two cohort studies of patients aged 66 or older conducted in the U.S.

For peer review only

and Canada found new postoperative prolonged opioid use at about 6% and 3%, respectively [4, 9]. Sun *et al.,* studied patients between the age of 18-64 and found prolonged opioid use at around 1% [15]. The discrepancy in occurrence of postoperative prolonged opioid use between these studies can at least partially be explained by differences in definitions of prolonged opioid use and how long a patient must be opioid naïve for. Sun *et al.,* used the most conservative definition of prolonged use where patients needed to fill 10 or more prescriptions or more than 120 days' supply of an opioid in the first year after surgery [15]. While this definition captures the patients with a high postsurgical opioid consumption it may underestimate the risk for patients with a lower, yet problematic, use. Although some patients will need opioids to manage postoperative acute pain, this period should be limited. Previous studies have shown that half of patients using opioids for longer than 90 days persist in their usage for many years after initiation [16, 17]. This highlights the importance of restrictiveness in prescribing opioids. Two of these studies only included privately insured patients [4, 15]. This introduces a bias in only selecting patients with the ability to afford a private insurance, potentially yielding a healthier population that is not generalizable to the actual population of the country. The lack of private insurances in a Swedish cohort is another strength of this study which adds additional insight to the field. Another aspect that could lead to differences in results between these studies is the lack of consensus regarding how to handle patients that died before the end of follow-up. We excluded these patients before analysis to avoid competing risk. In the Canadian cohort patients that were unable to meet the primary outcome were also excluded [9] whereas the three other studies do not state this [4, 6, 15].

Patient related risk factors for postoperative opioid use

The risk of prolonged opioid use was higher for women. This is not a consistent finding across the literature as other studies have found male sex [15] to be a greater risk factor, or no

For peer review only

significant gender difference [4, 8, 9]. For comorbidities, a higher CCI-score generated a higher aOR. We did not find a distinct trend regarding age where patients between 50-59 years of age had the highest aOR. Other studies have found both the oldest and the youngest population to be at higher risk, but also no significant difference in terms of age [4, 8, 9, 15]. History of psychiatric disease and/or medication led to almost a two-fold risk increase. Excluding the oldest population in a restriction analysis further increased the aOR for history of psychiatric disease indicating that the impact of psychiatric disease as a risk factor for new postoperative prolonged opioid use is stronger in younger patients (supplementary table 4). In line with this, other studies have found an association between preoperative benzodiazepine or antidepressant use, and prolonged postoperative opioid consumption [10, 18]. Our results, alongside aforementioned studies, indicate that clinicians must be aware of this relationship when prescribing postoperative opioids.

Surgery related risk factors for postoperative opioid use

Acute surgery had a higher occurrence of postoperative opioid use compared to elective surgery. Being exposed to cancer surgery yielded an aOR of 1.06 (95% CI: 1.02-1.14). A patient going through cancer surgery is a potential confounder in our study as treating cancer patients with opioids often is unavoidable. Partly, we tried to account for this by excluding patients prescribed opioids up to 180 days before surgery. Moreover, since individuals dying up to a year after surgery were excluded, we were hopefully able to account for unsuccessful surgical removal of the tumour and deterioration of advanced stages of malignancies. Our finding of a very low risk increase for cancer surgery speaks against this being a significant confounder.

For peer review only

Conclusion

In this large national observational cohort study, 7% developed new prolonged opioid use postoperatively. We identified several potential risk factors where a history of psychiatric disease was the most important. Data on these susceptible patients could help clinicians reduce the number of prolonged opioid users by adapting their analgesic and preventative strategies. Clinicians need be aware of the risks, have a plan for tapering, and realize that every opioid prescription has the potential of turning a non-user into a long-term user.

For peer review only

Data availability statement

Data may be obtained from a third party and are not publicly available. Data from Swedish health registers are available to all researchers upon making an application.

Ethics statements

Patient consent for publication

Not applicable.

Ethics approval

The study protocol was approved by the Regional Ethics Committee of Stockholm, Sweden (2014/1306-31/3).

For peer review only

Author Contributions

FL, MB and LH were responsible for the study concept and design. LH did the data collection and data cleaning. FL and LH did the data analysis. FL drafted the manuscript. MB, EL and LH did critical review of the manuscript. All the authors interpreted the data and approved the final manuscript as submitted and agree to be accountable for all aspects of the work.

Funding Statement

This research received no specific grant from any funding agency in the public, commercial or not-for-profit sectors.

Competing Interests Statement

None of the authors have any competing interests to declare.

For peer review only

References

[1] A. N. Nafziger and R. L. Barkin, "Opioid Therapy in Acute and Chronic Pain," (in eng), *J Clin Pharmacol,* vol. 58, no. 9, pp. 1111-1122, Sep 2018, doi: 10.1002/jcph.1276.

[2] T. G. Weiser *et al.*, "Estimate of the global volume of surgery in 2012: an assessment supporting improved health outcomes," (in eng), *Lancet,* vol. 385 Suppl 2, p. S11, Apr 27 2015, doi: 10.1016/s0140-6736(15)60806-6.

[3] C. E. Callinan, M. D. Neuman, K. E. Lacy, C. Gabison, and M. A. Ashburn, "The Initiation of Chronic Opioids: A Survey of Chronic Pain Patients," (in eng), *J Pain,* vol. 18, no. 4, pp. 360-365, Apr 2017, doi: 10.1016/j.jpain.2016.11.001.

[4] C. M. Brummett *et al.*, "New Persistent Opioid Use After Minor and Major Surgical Procedures in US Adults," (in eng), *JAMA Surg,* vol. 152, no. 6, p. e170504, Jun 21 2017, doi: 10.1001/jamasurg.2017.0504.

[5] M. J. Hartwell, R. S. Selley, M. A. Terry, and V. K. Tjong, "Can We Eliminate Opioid Medications for Postoperative Pain Control? A Prospective, Surgeon-Blinded, Randomized Controlled Trial in Knee Arthroscopic Surgery," (in eng), *Am J Sports Med,* vol. 48, no. 11, pp. 2711-2717, Sep 2020, doi: 10.1177/0363546520941861.

[6] A. Alam, T. Gomes, H. Zheng, M. M. Mamdani, D. N. Juurlink, and C. M. Bell, "Long-term analgesic use after low-risk surgery: a retrospective cohort study," (in eng), *Arch Intern Med,* vol. 172, no. 5, pp. 425-30, Mar 12 2012, doi: 10.1001/archinternmed.2011.1827.

[7] K. S. Ladha *et al.*, "Opioid Prescribing After Surgery in the United States, Canada, and Sweden," (in eng), *JAMA Netw Open,* vol. 2, no. 9, p. e1910734, Sep 4 2019, doi: 10.1001/jamanetworkopen.2019.10734.

[8] N. M. Edwards, C. Varnum, S. Overgaard, L. Nikolajsen, C. F. Christiansen, and A. B. Pedersen, "Risk factors for new chronic opioid use after hip fracture surgery: a Danish nationwide cohort study from 2005 to 2016 using the Danish multidisciplinary hip fracture registry," (in eng), *BMJ Open,* vol. 11, no. 3, p. e039238, Mar 8 2021, doi: 10.1136/bmjopen-2020-039238.

[9] H. Clarke, N. Soneji, D. T. Ko, L. Yun, and D. N. Wijeysundera, "Rates and risk factors for prolonged opioid use after major surgery: population based cohort study," (in eng), *BMJ,* vol. 348, p. g1251, Feb 11 2014, doi: 10.1136/bmj.g1251.

[10] T. J. Rajamäki, T. Moilanen, P. A. Puolakka, A. Hietaharju, and E. Jämsen, "Is the Preoperative Use of Antidepressants and Benzodiazepines Associated with Opioid and Other Analgesic Use After Hip and Knee Arthroplasty?," (in eng), *Clin Orthop Relat Res,* vol. 479, no. 10, pp. 2268-2280, Oct 1 2021, doi: 10.1097/corr.0000000000001793.

[11] J. F. Ludvigsson, P. Otterblad-Olausson, B. U. Pettersson, and A. Ekbom, "The Swedish personal identity number: possibilities and pitfalls in healthcare and medical research," (in eng), *Eur J Epidemiol,* vol. 24, no. 11, pp. 659-67, 2009, doi: 10.1007/s10654-009-9350-y.

[12] J. F. Ludvigsson *et al.*, "External review and validation of the Swedish national inpatient register," (in eng), *BMC Public Health,* vol. 11, p. 450, Jun 9 2011, doi: 10.1186/1471-2458-11-450.

[13] Socialstyrelsen. "Läkemedelsregistret." Socialstyrelsen. https://www.socialstyrelsen.se/statistik-och-data/register/lakemedelsregistret/ (accessed 2022-05-24, 2022).

[14] Socialstyrelsen. "Dödsorsaksregistret." https://www.socialstyrelsen.se/statistik-och-data/register/dodsorsaksregistret/ (accessed.

[15] E. C. Sun, B. D. Darnall, L. C. Baker, and S. Mackey, "Incidence of and Risk Factors for Chronic Opioid Use Among Opioid-Naive Patients in the Postoperative Period," *Jama Internal Medicine,* vol. 176, no. 9, pp. 1286-1293, Sep 2016, doi: 10.1001/jamainternmed.2016.3298.

[16] M. Von Korff *et al.*, "De facto long-term opioid therapy for noncancer pain," (in eng), *Clin J Pain,* vol. 24, no. 6, pp. 521-7, Jul-Aug 2008, doi: 10.1097/AJP.0b013e318169d03b.

For peer review only

[17] B. C. Martin, M. Y. Fan, M. J. Edlund, A. Devries, J. B. Braden, and M. D. Sullivan, "Long-term chronic opioid therapy discontinuation rates from the TROUP study," (in eng), *J Gen Intern Med,* vol. 26, no. 12, pp. 1450-7, Dec 2011, doi: 10.1007/s11606-011-1771-0.
[18] C. A. Rishel, Y. Zhang, and E. C. Sun, "Association Between Preoperative Benzodiazepine Use and Postoperative Opioid Use and Health Care Costs," (in eng), *JAMA Netw Open,* vol. 3, no. 10, p. e2018761, Oct 1 2020, doi: 10.1001/jamanetworkopen.2020.18761.

For peer review only

Figure legends

Figure 1. Participant flowchart

Figure 1. Risk factors for developing prolonged opioid use after surgery among patients with no opioid use 180 days before surgery. Odds ratios adjusted for sex, age, comorbidities (charlson comorbidity index) and surgical procedure.

** A diagnosis of ≥ 1 of the psychiatric diseases listed (Table 1) and/or collected a prescription of ≥ of the listed psychiatric medications (Table 1) within five years of surgery.

For peer review only

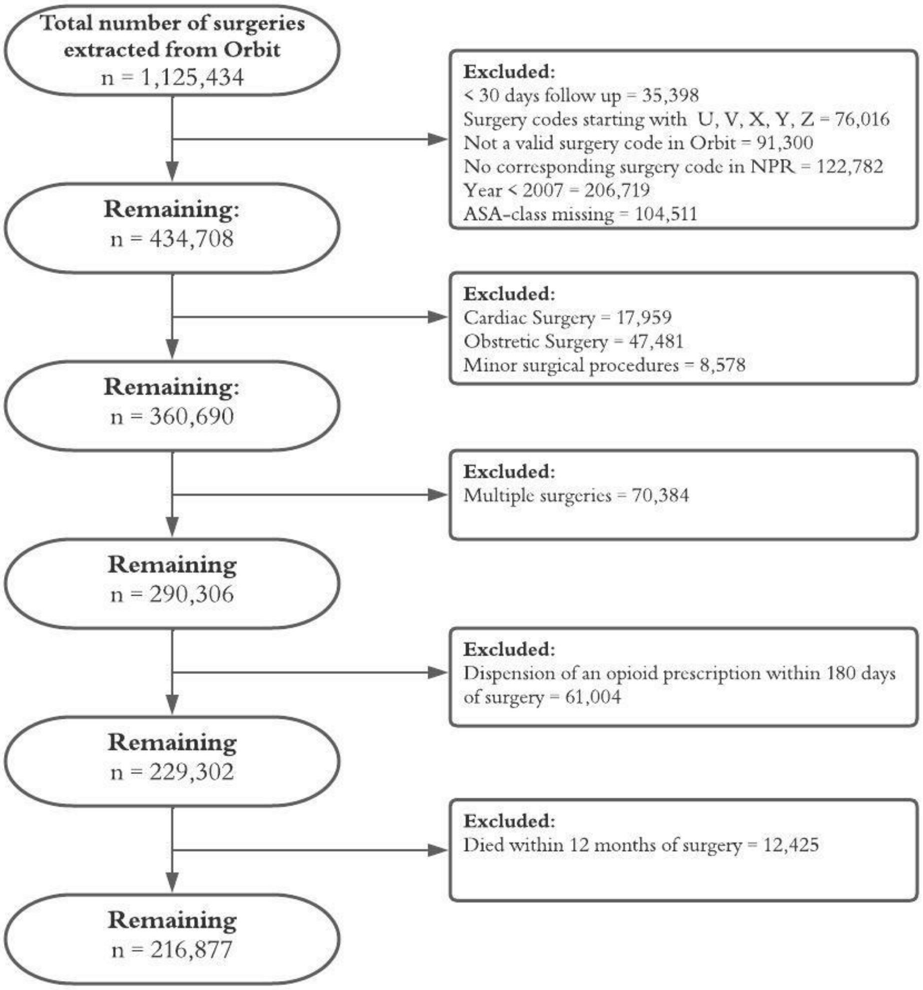

Figure 1. Participant flowchart

142x152mm (160 x 160 DPI)

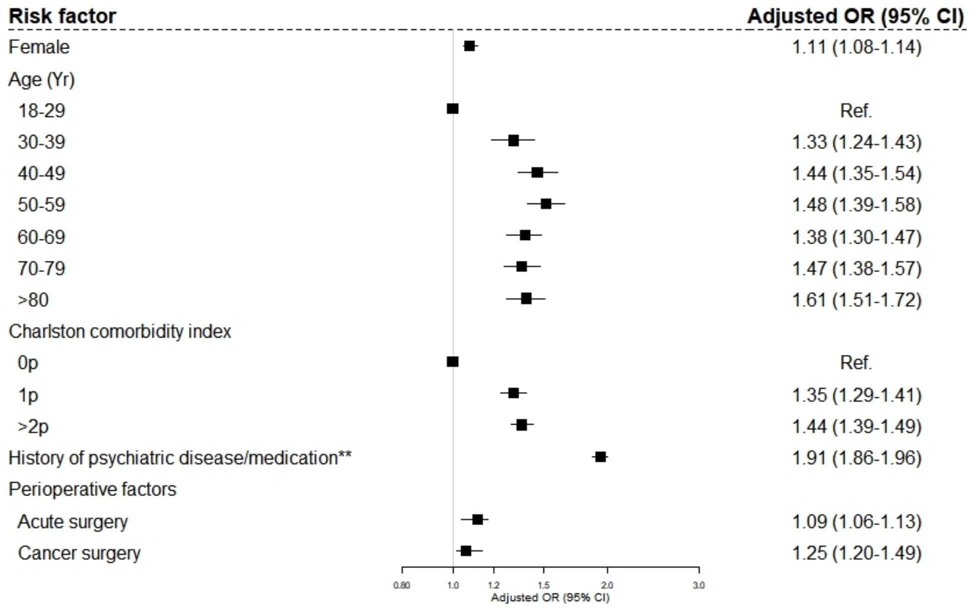

Figure 2. Risk factors for developing prolonged opioid use after surgery among patients with no opioid use 180 days before surgery. Odds ratios adjusted for sex, age, comorbidities (charlson comorbidity index) and surgical procedure.
** A diagnosis of ≥ 1 of the psychiatric diseases listed (Table 1) and/or collected a prescription of ≥ of the listed psychiatric medications (Table 1) within five years of surgery.

228x158mm (96 x 96 DPI)

Supplementary table 1: Included and excluded surgery codes.

Included surgery codes:

A – nervous system
B – endocrine system
C – eye and adjacent structures
D - ear, nose, and throat
G - pulmonary
H – mammary gland
J - gastrointestinal
K - urology
L - gynecology
N - musculoskeletal system
P - vascular
Q – dermatology

Excluded surgery codes:

F – cardiac
M – obstetric
T – minor surgical procedures
U – transluminal endoscopy
V – Surgery on aorta, peripheral vessels, and lymphatic system.
X – diagnostic procedures associated with surgery
Y – removal of organs for transplant
Z – additional codes

Minor surgical procedures include:

Neurological procedures: lumbar puncture, peripheral nerve block, sympathetic block TAW and other minor neurosurgical procedure.

Endocrine surgery: puncture of thyroid gland, needle biopsy of thyroid gland, aspiration cytology of thyroid gland, puncture of parathyroid gland, needle biopsy of parathyroid gland, needle biopsy of adrenal gland, aspiration cytology of adrenal gland, other minor procedure in endocrine surgery

Eye surgery: needle biopsy of orbit, aspiration cytology of orbit, retrobulbar therapeutic injection into orbit, peribulbar therapeutic injection into orbit, needle biopsy of eyelid, simple epilation of cilia, therapeutic injection into eyelid, removal of ptosis suture from eyelid, adjustment of ptosis suture, needle biopsy of lacrimal gland, probing of lacrimal canaliculus or of nasolacrimal duct, intubation of lacrimal canaliculus or intubation of nasolacrimal duct, therapeutic injection into extraocular muscle, subconjunctival therapeutic injection, protection of cornea and sclera with soft lens, removal of sutures from cornea, laser suture lysis in cornea or sclera, needle aspiration of aqueous from anterior chamber, needle biopsy of iris, needle biopsy of ciliary body, needle biopsy of choroid, needle biopsy of vitreous body, needle biopsy of retina, other minor procedure in eye surgery

Ear, nose and throat: biopsy of auricle or external auditory canal, needle biopsy of auricle or external auditory canal, removal of foreign body from external auditory canal, needle biopsy of tympanic membrane, needle biopsy of nose, removal of foreign body from cavity of nose, needle biopsy of septum of nose, needle biopsy of maxillary antrum, puncture and irrigation of maxillary antrum, needle biopsy of ethmoidal bone and sinus, needle biopsy of frontal sinus, needle biopsy of sphenoidal sinus, needle biopsy of larynx, other minor procedure in surgery of ear, nose and larynx

For peer review only

Breast surgery: needle biopsy of mamilla, needle biopsy of mammary gland, other minor procedure in surgery of mammary gland

Gastroenterological surgery: puncture of peritoneum, laparocentesis, percutaneous peritoneal irrigation, percutaneous introduction of peritoneal dialysis catheter, removal of peritoneal dialysis catheter, percutaneous local drainage of peritoneal cavity, introduction of intraperitoneal injection port, removal of intraperitoneal injection port, insertion of ballon tube for tamponade of oesophageal varices, insertion of nasogastric or naso-gastroduodenal tube, insertion of other tube into stomach or duodenum, change of gastrostomy tube, insertion of jejunal tube, insertion of jejunal tube through gastrostomy, peroral biopsy of small intestine, reduction of intestinal intussusception using barium enema, digital extraction of impacted faeces, removal of foreign body from rectum, percutaneous needle biopsy of liver, percutaneous destruction of lesion of liver, intraoperative cholangiography, laparoscopic cholangiography, percutaneous needle biopsy of pancreas, percutaneous drainage of pseudocyst or abscess of pancreas, needle biopsy of spleen, other minor procedure in gastroenterological surgery.

Urology: needle biopsy of kidney or pelvis of kidney, aspiration cytology of kidney, percutaneous puncture of kidney or pelvis of kidney, needle biopsy of bladder, percutaneous puncture of bladder, catheterisation of bladder, dilatation of urethra, needle biopsy of prostate, aspiration cytology of prostate, puncture of prostate, needle biopsy of testis, aspiration cytology of testis, puncture of testis, needle biopsy of epididymis, aspiration cytology of epididymis, puncture of epididymis, puncture of scrotum, other minor surgical procedure in urology

Gynecology: needle biopsy of ovary, needle biopsy of Fallopian tube, insertion of intrauterine contraceptive device, dilatation of vagina, removal of foreign body from vagina in childhood, transvaginal puncture of cul-de-sac, other minor surgical procedure in gynaecology

Orthopedic surgery: puncture or needle biopsy of soft tissue, simple incision of soft tissue, arthrocentesis, injection of diagnostic or therapeutic substance into joint, aspiration of bone marrow, drill biopsy of bone marrow, soft bandage, prefabricated orthosis, custom moulded splint, custom moulded cast, major custom moulded cast, other orthopedic bandage, external traction, implantation of skeletal marker

Peripheral vessels and lymphatic system: phlebotomy, open cannulation of peripheral vein, sclerotherapy of varicose veins, insertion of central venous catheter through external or internal jugular vein, insertion of central venous catheter through subclavian or brachiocephalic vein, biopsy of superficial lymph node, needle biopsy of lymph node, other minor surgical procedure on peripheral vessel or lymphatic system, biopsy of artery or vein, implantation of vascular injection port, removal of vascular injection port

Dermatology: other minor procedure on skin, biopsy of skin, needle biopsy of skin, removal of sutures of skin, adjustment of volume of tissue expander

For peer review only

Supplementary table 2. Risk factors for developing prolonged opioid use after surgery among patients with no opioid use 180 days before surgery. Odds ratios adjusted for sex, age and surgical procedure.

Risk factor	Crude OR (95% CI)	Adjusted OR (95% CI)[1]
**Patient factors**		
Female	1.08 (1.05-1.12)	1.16 (1.12-1.21)
Age (yr) 18-29	Ref.	Ref.
30-39	1.31 (1.19-1.44)	1.43 (1.30-1.57)
40-49	1.52 (1.40.-1.66)	1.67 (1.53-1.82)
50-59	1.90 (1.75-2.06)	1.91 (1.76-2.07)
60-69	1.96 (1.82-2.11)	1.85 (1.71-2.00)
70-79	2.19 (2.03-2.36)	1.92 (1.77-2.07)
> 80	2.80 (2.59-3.03)	2.16 (1.99-2.34)
**Preoperative data**		
Charlson comorbidity index 0p	Ref.	Ref.
1p	1.65 (1.56-1.74)	1.43 (1.35-1.51)
≥ 2p	1.42 (1.37-1.47)	1.69 (1.63-1.76)
ASA classification 1	Ref.	Ref.
2	1.83 (1.76-1.92)	1.65 (1.57-1.73)
3	2.57 (2.45-2.69)	2.25 (2.13-2.38)
4	3.07 (2.71-3.47)	2.76 (2.43-3.14)
Comorbidities		
Heart disease[†]	1.54 (1.48-1.59)	1.32 (1.27-1.37)
Lung disease[‖]	1.79 (1.69-1.91)	1.59 (1.49-1.70)
Renal disease[‡]	1.35 (1.24-1.48)	1.46 (1.33-1.60)
Diabetes mellitus	1.53 (1.46-1.61)	1.40 (1.33-1.47)
Vascular disease[↑]	1.44 (1.32-1.56)	1.44 (1.32-1.57)
Cerebrovascular disease[§]	1.50 (1.40-1.62)	1.25 (1.16-1.35)
History of psychiatric disease		
Substance abuse[¶]	1.97 (1.83-2.13)	1.99 (1.84-2.15)
Cognitive disease[ǂ]	2.15 (1.96-2.36)	1.50 (1.36-1.65)
Personality disorder, Schizophrenia[¥]	1.23 (1.12-1.35)	1.52 (1.38-1.67)
Affective disorder[£]	1.63 (1.52-1.75)	1.70 (1.59-1.83)
Anxiety disorder[®]	1.58 (1.47-1.69)	1.81 (1.68-1.95)
Preoperative Medication[♦]		
Neuroleptics	1.75 (1.63-1.89)	1.58 (1.46-1.71)
Benzodiazepines	1.84 (1.77-1.91)	1.75 (1.68-1.82)
Hypnotics & Sedatives	2.11 (2.04-2.19)	1.95 (1.89-2.02)
Antidepressants	1.90 (1.83-1.97)	1.80 (1.73-1.86)
Psychostimulants	1.70 (1.44-2.01)	2.18 (1.84-2.58)
History of psychiatric disease and/or medication**	2.10 (2.03-2.17)	1.98 (1.92-2.05)
**Perioperative factors**		
Acute surgery*	1.35 (1.30-1.39)	1.04 (1.01-1.09)
Cancer surgery	1.10 (1.05-1.14)	1.08 (1.04-1.14)

† Chronic ischemic heart disease, Angina pectoris, Hypertensive disease, Cardiac arrest, Heart failure, Valve disease, Pulmonary heart disease, Cardiomyopathy, Conduction disorders/Cardiac arrhythmias, Cardiac arrest, Diseases of arteries, arterioles and capillaries.

|| Pneumonia, COPD.

‡ Acute renal failure/unspecified renal failure, Chronic renal failure, Other renal disease.

↑Atherosclerosis, Aortic aneurysm and dissection, Other aneurysm, Other peripheral vascular diseases, Arterial embolism and thrombosis, Atheroembolism, Septic arterial embolism, Other disorders of arteries and arterioles, Diseases of capillaries, Disorders of arteries, arterioles and capillaries in diseases classified elsewhere.

§ Subarachnoid hemorrhage, Intracerebral hemorrhage, Other nontraumatic intracranial haemorrhage, Cerebral infarction, Acute cerebrovascular disease without cerebral infarction, Vascular occlusion without cerebral infarction, Other cerebrovascular diseases, Cerebrovascular disorders in diseases classified elsewhere, Sequelae of cerebrovascular disease.

¶ Mental and behavioural disorders due to psychoactive substance use.

ǂ Alzheimer's disease, Vascular dementia, Other dementia, Unspecified dementia, Non-alcoholic amnesia, Non-alcoholic delirium, mental disorders due to known physiological condition, Personality and behavioral disorders due to known physiological condition, mental disorder due to unknown somatic or organic disorder.

¥ Schizophrenia, Schizotypal disorder, Persistent delusional disorders, Acute and transient psychotic disorders, Induced delusional disorder, Schizoaffective disorders, Other nonorganic psychotic disorders, Unspecified nonorganic psychosis.

£ Manic episode, bipolar affective disorder, depressive episode, recurrent depressive disorder, persistent mood disorders, other affective mood disorders, unspecified affective mood disorders.

® Phobic anxiety disorders, other anxiety disorders, obsessive-compulsive disorder, reaction to severe stress and adjustment disorders, dissociative disorders, somatoform disorders, other neurotic disorders.

Ͱ Cognitive disease, substance abuse disorder, personality disorder/schizophrenia, affective disorder, anxiety disorder, Psychiatric medication within 5 years of surgery..

♦ Collection of prescription within five years of surgery.

* Acute surgery is defined as procedures that are admitted as acute surgeries. The level of acuteness can differ between right away, 6h and 72h depending on type of procedure.

** A diagnosis of ≥ 1 of the psychiatric diseases listed (Table 1) and/or collected a prescription of ≥ of the listed psychiatric medications (Table 1) within five years of surgery.

For peer review only

Supplementary table 3. *Baseline characteristics of surgical cohort at inclusion (n=216,877) and proportion of new prolonged opioid consumption.*

		New Prolonged Opioid Consumption		
	Total n = 216 877 (%)	No n = 201 796 (93)	Yes n = 15 081 (7.0)	*p*-value[2,3]
**Procedure – no (%[1])**				<0.001
Ophthalmic surgery	3 228	3 167 (98)	611 (1.9)	
Neurosurgery	14 052	13 019 (93)	1 033 (7.4)	
Endocrine surgery	7 573	7 429 (98)	144 (1.9)	
Ear, nose & throat surgery	6 839	6 666 (97)	173 2.5)	
Oral & maxillofacial surgery	8 729	8 331 (95)	398 (4.6)	
Pulmonary surgery	2 244	2 023 (90)	221 (9.9)	
Breast surgery	12 635	12 104 (96)	531 (3.5)	
Gastrointestinal surgery	43 112	40 521 (94)	2 591 (6)	
Urologic surgery	23 642	22 509 (95)	1 133 (4.8)	
Gynecologic surgery	18 994	18 412 (97)	582 (3.1)	
Orthopedic surgery	61 360	54 003 (88)	7 357 (12)	
Vascular surgery	8 450	7 965 (94)	485 (5.7)	
Dermatologic surgery	6 019	5 647 (94)	372 (6.2)	
**Year of surgery – year (%[1])**				0.52
2007-2008	42 195	39 192 (93)	5 239 (7.1)	
2009-2010	46 963	43 705 (93)	5 641 (6.9)	
2011-2012	60 213	56 067 (93)	7 174 (6.9)	
2013-2014	67 506	62 832 (93)	7 943 (6.9)	
**Region of surgery – no (%[1])**				0.009
Stockholm & Södermanland	61 142	56 935 (93)	4 207 6.9)	
Skåne	80 964	75 147 (93)	5 817 (7.2)	
Västra Götaland	37 090	34 566 (93)	2 524 (6.8)	
Västernorrland & Västerbotten	37 681	35 148 (93)	2 533 (6.7)	

[1] Percentages calculated in relation to horizontal study cohort, [2] Pearson's Chi-squared test, [3] Mann-Whitney U-test

Supplementary table 4. Sensitivity analyses of the association between history of psychiatric disease and prolonged opioid use. Restriction of the cohort; excluding patients >70 years old (75th percentile).

Risk factor	OR* (95% CI)
History of psychiatric disease	2.07 (1.99 to 2.17)

* Adjusted for sex, age, comorbidities (charlson comorbidity index) and surgical procedure.

For peer review only

STROBE Statement—Checklist of items that should be included in reports of ***cohort studies***

	Item No	Recommendation	Page No
**Title and abstract**	1	(*a*) Indicate the study's design with a commonly used term in the title or the abstract	1
		(*b*) Provide in the abstract an informative and balanced summary of what was done and what was found	1
**Introduction**			
Background/rationale	2	Explain the scientific background and rationale for the investigation being reported	3
Objectives	3	State specific objectives, including any prespecified hypotheses	3-4
**Methods**			
Study design	4	Present key elements of study design early in the paper	5-6
Setting	5	Describe the setting, locations, and relevant dates, including periods of recruitment, exposure, follow-up, and data collection	5
Participants	6	(*a*) Give the eligibility criteria, and the sources and methods of selection of participants. Describe methods of follow-up	5
		(*b*) For matched studies, give matching criteria and number of exposed and unexposed	N/A
Variables	7	Clearly define all outcomes, exposures, predictors, potential confounders, and effect modifiers. Give diagnostic criteria, if applicable	6-7
Data sources/ measurement	8*	For each variable of interest, give sources of data and details of methods of assessment (measurement). Describe comparability of assessment methods if there is more than one group	5-6
Bias	9	Describe any efforts to address potential sources of bias	8
Study size	10	Explain how the study size was arrived at	Figure 1
Quantitative variables	11	Explain how quantitative variables were handled in the analyses. If applicable, describe which groupings were chosen and why	7
Statistical methods	12	(*a*) Describe all statistical methods, including those used to control for confounding	7
		(*b*) Describe any methods used to examine subgroups and interactions	7
		(*c*) Explain how missing data were addressed	7
		(*d*) If applicable, explain how loss to follow-up was addressed	N/A
		(*e*) Describe any sensitivity analyses	10
**Results**			
Participants	13*	(a) Report numbers of individuals at each stage of study—eg numbers potentially eligible, examined for eligibility, confirmed eligible, included in the study, completing follow-up, and analysed	Figure 1
		(b) Give reasons for non-participation at each stage	5 & Figure 1
		(c) Consider use of a flow diagram	Figure 1
Descriptive data	14*	(a) Give characteristics of study participants (eg demographic, clinical, social) and information on exposures and potential confounders	5, 9 & Table 1
		(b) Indicate number of participants with missing data for each variable of interest	Table 1

For peer review only - http://bmjopen.bmj.com/site/about/guidelines.xhtml

		(c) Summarise follow-up time (eg, average and total amount)	6
Outcome data	15*	Report numbers of outcome events or summary measures over time	9 & Table 1
Main results	16	(*a*) Give unadjusted estimates and, if applicable, confounder-adjusted estimates and their precision (eg, 95% confidence interval). Make clear which confounders were adjusted for and why they were included	9, 10 & Table 1
		(*b*) Report category boundaries when continuous variables were categorized	7-8
		(*c*) If relevant, consider translating estimates of relative risk into absolute risk for a meaningful time period	
Other analyses	17	Report other analyses done—eg analyses of subgroups and interactions, and sensitivity analyses	10
**Discussion**			
Key results	18	Summarise key results with reference to study objectives	9, 10
Limitations	19	Discuss limitations of the study, taking into account sources of potential bias or imprecision. Discuss both direction and magnitude of any potential bias	14, 15
Interpretation	20	Give a cautious overall interpretation of results considering objectives, limitations, multiplicity of analyses, results from similar studies, and other relevant evidence	11-13
Generalisability	21	Discuss the generalisability (external validity) of the study results	13-14
**Other information**			
Funding	22	Give the source of funding and the role of the funders for the present study and, if applicable, for the original study on which the present article is based	16

*Give information separately for exposed and unexposed groups.

Note: An Explanation and Elaboration article discusses each checklist item and gives methodological background and published examples of transparent reporting. The STROBE checklist is best used in conjunction with this article (freely available on the Web sites of PLoS Medicine at http://www.plosmedicine.org/, Annals of Internal Medicine at http://www.annals.org/, and Epidemiology at http://www.epidem.com/). Information on the STROBE Initiative is available at http://www.strobe-statement.org.

For peer review only

BMJ Open

New prolonged opioid consumption after major surgery in Sweden: a population-based retrospective cohort study

Journal:	*BMJ Open*
Manuscript ID	bmjopen-2022-071135.R2
Article Type:	Original research
Date Submitted by the Author:	06-Apr-2023
Complete List of Authors:	Lindeberg, Felix; Karolinska University Hospital, Department of Anaesthesia and Intensive Care Medicine; Karolinska Institute, Department of Physiology and Pharmacology<br>Bell, Max; Karolinska Institutet, Department of Physiology & Pharmacology; Karolinska University Hospital, Department of Physiology & Pharmacology<br>Larsson, Emma; Karolinska University Hospital, Department of Anaesthesia and Intensive Care Medicine; Karolinska Institute, Department of Physiology and Pharmacology<br>Hallqvist, Linn; Karolinska University Hospital, Department of Anaesthesia and Intensive Care Medicine; Karolinska Institute, Department of Physiology and Pharmacology
**Primary Subject Heading**:	Surgery
Secondary Subject Heading:	Medical management, Anaesthesia
Keywords:	SURGERY, ANAESTHETICS, PAIN MANAGEMENT, Substance misuse < PSYCHIATRY, REGISTRIES, Adult surgery < SURGERY

SCHOLARONE™
Manuscripts

I, the Submitting Author has the right to grant and does grant on behalf of all authors of the Work (as defined in the below author licence), an exclusive licence and/or a non-exclusive licence for contributions from authors who are: i) UK Crown employees; ii) where BMJ has agreed a CC-BY licence shall apply, and/or iii) in accordance with the terms applicable for US Federal Government officers or employees acting as part of their official duties; on a worldwide, perpetual, irrevocable, royalty-free basis to BMJ Publishing Group Ltd ("BMJ") its licensees and where the relevant Journal is co-owned by BMJ to the co-owners of the Journal, to publish the Work in this journal and any other BMJ products and to exploit all rights, as set out in our licence.

The Submitting Author accepts and understands that any supply made under these terms is made by BMJ to the Submitting Author unless you are acting as an employee on behalf of your employer or a postgraduate student of an affiliated institution which is paying any applicable article publishing charge ("APC") for Open Access articles. Where the Submitting Author wishes to make the Work available on an Open Access basis (and intends to pay the relevant APC), the terms of reuse of such Open Access shall be governed by a Creative Commons licence – details of these licences and which Creative Commons licence will apply to this Work are set out in our licence referred to above.

Other than as permitted in any relevant BMJ Author's Self Archiving Policies, I confirm this Work has not been accepted for publication elsewhere, is not being considered for publication elsewhere and does not duplicate material already published. I confirm all authors consent to publication of this Work and authorise the granting of this licence.

For peer review only

New prolonged opioid consumption after major surgery in Sweden: a population-based retrospective cohort study

Felix Lindeberg[1,2], B.Sc; Max Bell[1,2], MD, PhD; Emma Larsson[1,2], MD, PhD; Linn Hallqvist[1,2], MD, PhD.

[1] The Department of Anaesthesia and Intensive Care Medicine, Karolinska University Hospital, Stockholm, Sweden
[2] The Department of Physiology and Pharmacology, Karolinska Institute, Stockholm, Sweden.

Corresponding author:

Felix Lindeberg

Felix.lindeberg@stud.ki.se
+46767847468
Dept of Perioperative Medicine and Intensive care (PMI)
Karolinska University Hospital, Solna
Eugeniavägen 3, 171 76 STOCKHOLM
SWEDEN

Manuscript word count: 2898

For peer review only

Abstract

Objective: Given that long term opioid usage is an important problem worldwide and postsurgical pain is a common indication for opioid prescription our primary objective was to describe the frequency of new prolonged opioid consumption after major surgery in Sweden, and, secondly, to evaluate potential associated risk factors.

Design: Cohort study including data from January 1, 2007, to December 31, 2014. Data regarding surgical procedures, baseline characteristics and outcomes was retrieved from the Orbit surgical planning system, the Swedish national patient register and the Swedish cause of death register.

Setting: Observational multicentre cohort study with data from 23 Swedish hospitals.

Participants: We included 216,877 patients aged ≥18 years, undergoing non-cardiac surgery, not exposed to opioids 180 days before, and alive 12 months after surgery.

Primary and secondary outcome measures: The primary endpoint was collection of at least three opioid prescriptions during the first postoperative year; within 90 days, day 91-180 and 181-365 after surgery in a previously opioid naïve patient. Secondly, multivariable logistic regression analysis was conducted to explore potential risk factors associated with prolonged opioid use.

Results: Of the 216,877 patients identified to undergo analysis, 15,081 (7.0%) developed new prolonged opioid consumption. Several risk factors were identified. Having a history of psychiatric disease was identified as the strongest risk factor (aOR: 1.94. 95% CI: 1.87-2.00).

Conclusion: In a large Swedish cohort of surgical patients, 7% developed new prolonged opioid consumption after major surgery. Our data on susceptible patients could help clinicians reduce the number of prolonged opioid users by adapting their analgesic and preventative strategies.

For peer review only

Strengths and limitations of this study

- A nationwide observational cohort study investigating opioid prescription after major surgery on a large Swedish population.
- The study cohort include adults of all ages, exposed to various major surgeries, and data is collected from Swedish health registers which offers robustness and great coverage.
- The lack of consensus in defining prolonged opioid consumption is a weakness of our study and others in the field.
- This study does not portray patient compliance to their prescription as dispensing of opioid prescriptions is a proxy for opioid consumption.
- Lack of long-term follow up, after the first postoperative year.

For peer review only

Introduction

The analgesic effect of opioids is a cornerstone of modern medicine but also fraught with side effects ranging from constipation and nausea to substance abuse and death [1]. More than 300 million people undergo surgery in the world every year and postsurgical pain is a common indication for opioid treatment [2, 3]. Many patients are exposed to opioids for the first time due to surgery and it has been suggested that postsurgical long-term opioid use should be referred to as a surgical complication [4]. In addition, opioids are often overprescribed after surgery and many patients report taking opioids for a different indication than for which the opioids were initially prescribed for [3, 5]. A previous study has indicated that patients who are prescribed opioids seven days after surgery are 50% more likely to collect an opioid prescription one year after surgery [6]. This is interesting since the proportion of patients receiving an opioid prescription at seven and 30 days after surgery differs enormously between countries [7].

Treating postsurgical pain with opioids, poses a challenge for physicians as two competing interests need to be addressed: managing acute pain and minimizing the risk of prolonged opioid use. A recent study showed that a high percentage of patients with a hip fracture, opioid naïve before surgery, were still using opioids several months after their surgical procedure [8]. Furthermore, risk factors for prolonged opioid use have been identified including type of surgery, comorbidities, and use of specific drugs ahead of surgery, e.g., antidepressants, and benzodiazepines [8-10]. Although some data exists, preoperative risk assessment might stand to gain from more data on risk factors of prolonged opioid use.

Therefore, the primary aim of the study was to describe the frequency of new prolonged opioid consumption after major surgery in Sweden. Data from a Swedish cohort would add insight to the field as most of similar studies have been conducted on North American

For peer review only

cohorts. Secondly, we sought to evaluate the impact of patient factors (age, sex, medical history), surgical factors (procedure, medical treatment) and healthcare system related factors (regional differences and time trends). Based on earlier studies from other countries we hypothesized that differences associated with both patient- and surgical characteristics exist.

For peer review only

Materials and Methods

Study Design & Participants

This was an observational multicentre cohort study using data from 23 Swedish hospitals at university-, county-, and district level, covering approximately 40 percent of the Swedish perioperative care. Included hospitals are located in different legislative regions and dispersed geographically throughout the country which makes the data generalizable to the whole Swedish population. Patients aged ≥ 18 years, undergoing surgery from January 1, 2007, to December 31, 2014, were included. Exclusion criteria were: collection of an opioid prescription within 180 days ahead of surgery, not being alive 12 months after surgery, undergoing cardiac, obstetric, ambulatory, minor (supplementary table 1), or multiple surgeries as well as missing a valid surgery code in Orbit or a corresponding surgery code in National Patient Register (NPR). Additionally, patients identified from hospitals with a high proportion of missing American Society of Anaesthesiologists (ASA) physical status classification were also excluded (Figure 1). The opioid naïve period of 180 days aims to exclude patients with a persistent opioid consumption while still including the patients where surgery may act as the trigger for prolonged opioid consumption. The study protocol was approved by the Regional Ethics Committee of Stockholm, Sweden (2014/1306-31/3).

For peer review only

Data sources

Orbit - surgical planning system

Orbit is a surgical planning system used by Swedish hospitals and provides information about patient characteristics, ASA physical status classification, date, type and duration of surgery and anaesthesia, all linked to the Swedish personal identification number. Surgery cannot be

performed without admitting a patient to orbit. The system was used to identify the study cohort.

Health Registers

Data extracted from Orbit were linked to the (NPR) using the unique Swedish personal identification number (PIN) assigned at birth or immigration [11]. The NPR provides information of all hospital admissions since the year of 1987 and outpatient care since 2001 [12]. The Swedish prescribed drug register contains information about all dispensed *and collected* drug prescriptions in Sweden since July 2005 [13]. In the Swedish cause of death register virtually all (> 99%) deaths are registered since 1952 based on the current ICD-classification [14].

Data collection

Data was retrieved from the above-mentioned registers. Data collection included information about patient characteristics and medical history such as age, sex, ASA physical status classification, hospital diagnoses and collected drug prescriptions. This was made possible by backtracking the NPR and the Swedish prescribed drug register for five years before surgery and one year after surgery. Data from the Swedish cause of death register was collected for one year after surgery. Perioperative characteristics included date, type, and duration of surgery and was extracted from Orbit. Surgical procedures were clustered into twelve subtypes based on surgical codes used in the Nordic countries (NOMESCO). Subtypes included gastrointestinal; endocrine; ophthalmic; ear, nose, and throat; thoracic; neuro; breast; urologic; gynaecologic; orthopaedic; vascular; and dermatologic surgery.

For peer review only

Primary endpoint

The primary endpoint was new prolonged opioid use, defined as collection of at least three opioid prescriptions during the first postoperative year; within 90 days, day 91-180 and 181-365 after surgery in a previously opioid naïve patient. Being opioid naïve was defined as not collecting an opioid prescription within 180 days prior to surgery.

Secondary Aims

Secondary aims were to evaluate the impact of patient-, surgical-, and healthcare system related risk factors for prolonged opioid prescription in the opioid naïve patient. Patient risk factors included age, sex, as well as somatic and psychiatric comorbidities, surgical risk factors include type of surgery and medical treatment, whereas healthcare related risk factors are differences associated with four different regions in Sweden and the year of surgery.

Statistical Analysis

The data was analysed during the autumn of 2022. The study cohort was divided into two groups based on meeting the primary outcome or not. Categorical variables within each of these groups are presented as percentages of the total population and continuous data as medians with interquartile ranges (IQR) that represent 25-75th percentiles. Continuous variables were analysed using the Mann-Whitney U test and categorical variables were compared with the Chi-squared test generating p-values to look for statistical differences between the groups. P-values < 0.05 were considered significant and statistical tests were

For peer review only

two-sided. Logistic regression analysis was conducted to investigate the relationships between potential pre- and perioperative, patient-, and surgery-related risk factors and the associated effect on our primary endpoint. Variables with univariate significance, and considered clinically relevant, were explored in the multivariate analysis to explore our secondary aims, results showed in a supplementary table 2. Hence, crude and adjusted odds ratios, presented with 95% confidence intervals, were calculated to see how the risk estimates changed when adjusting for potential confounders. Adjustments for age, sex, comorbidities, year of surgery and type of surgery (including cancer surgery) was performed in the final model. Patients that died within one year of surgery were excluded due to competing risk. A sensitivity analysis including these patients were conducted which had little effect on the results. A history of psychiatric disease was significantly associated with elevated risk of prolonged opioid consumption, and we hypothesized that the impact of this risk factor might be different depending on age. Therefore, a restriction analysis was performed, using the adjusted model with exclusion of patients >70 years, in the upper 4th quartile (n=67 132). Data was analysed by STATA version 14.2 (Stata Corp., College Station, TX, USA).

Patient and Public Involvement

It was not possible to involve patients or the public in the design, or conduct, or reporting, or dissemination plans of our research.

For peer review only

Results

Patient characteristics

216,877 patients were identified to undergo analysis (Figure 1). 61,004 patients were excluded due to collection of an opioid prescription within 180 days of surgery and 12,425 patients were excluded because they died within 12 months of surgery (Figure 1). The population consisted of 118,698 (55%) women and had a median (IQR) age of 62 (45, 72) years old (Table 1). 21,169 (10%) of patients had a history of psychiatric disease and 78,743 (36%) had collected a prescription for psychiatric medication up to five years before surgery (Table 1). 152,204 (70%) of the conducted surgeries were elective and orthopaedic surgery was the most common procedure (Table 1).

For peer review only

Table 1. Baseline characteristics of surgical cohort at inclusion (n=216 877) and proportion of new prolonged opioid consumption

		New Prolonged Opioid Consumption		
	Total n = 216 877 (%)	No n = 201 796 (93)	Yes n = 15 081 (7.0)	*p*-value[2,3]
Age – median years (IQR)	62 (45, 72)	61 (44, 72)	66 (52, 76)	<0.001
Women – no. (%[1])	118 698 (55)	110 173 (55)	8 525 (57)	<0.001
**Age range – no. (%[1])**				<0.001
Age 18-29	22 996 (11)	22 118 (11)	878 (5.8)	
Age 30-39	18 509 (8.5)	17 593 (8.7)	916 (6.1)	
Age 40-49	25 634 (12)	24 171 (12)	1 463 (9.7)	
Age 50-59	31 973 (15)	29 732 (15)	2 241 (15)	
Age 60-69	49 472 (23)	45 894 (23)	3 578 (24)	
Age 70-79	41 394 (19)	38 989 (19)	3 314 (22)	
Age >80	26 899 (12)	24 208 (12)	2 691 (18)	
**Preoperative data – no. (%[1])**				
ASA Classification				<0.001
ASA1	74 067 (34)	70 947 (35)	3 120 (21)	
ASA2	96 381 (44)	89 173 (44)	7 208 (48)	
ASA3	43 853 (20)	39 406 (20)	4 447 (29)	
ASA4	2 576 (1.2)	2 270 (1.1)	306 (2.0)	
Charlson comorbidity index				<0.001
0	125 868 (58)	118 447 (59)	7 421 (49)	
1	18 408 (8.5)	16 684 (8.3)	1 724 (11)	
>2	72 601 (33)	66 319 (33)	10 751 (41)	
Heart disease[†]	51 693 (24)	46 904 (23)	4 796 (32)	<0.001
Lung disease[‖]	10 457 (4.8)	9 259 (4.6)	1 198 (7.9)	<0.001
Renal disease[‡]	6 079 (2.8)	5 525 (2.7)	554 (3.7)	<0.001
Diabetes Mellitus	19 630 (9.0)	17 694 (8.8)	1 936 (13)	<0.001
Peripheral vascular disease[↑]	6 837 (3.2)	6 182 (3.1)	655 (4.3)	<0.001
Cerebrovascular disease[§]	8 323 (3.8)	7 496 (3.7)	827 (5.5)	<0.001
Cognitive disease[ᵻ]	3 908 (1.8)	3 375 (1.7)	533 (3.5)	<0.001
Substance abuse disease[¶]	6 191 (2.9)	5 413 (2.7)	788 (5.2)	<0.001
Personality disorder, schizophrenia[¥]	5 795 (2.7)	5 310 (2.6)	485 (2.6)	<0.001
Affective disorders[£]	8 435 (3.9)	7 536 (3.7)	899 (6.0)	<0.001
Anxiety disorders[®]	8 774 (4.1)	7 867 (3.9)	907 (6.0)	<0.001
History of psychiatric disease[▱]	21 169 (10)	19 056 (9.4)	2 113 (14)	<0.001
**Preoperative medication – no (%[1])[♦]**				
Psychiatric medication[Ψ]	78 743 (36)	70 692 (35)	8 051 (53)	<0.001
Neuroleptics	7 007 (3.2)	6 211 (3.1)	796 (5.3)	<0,001
Benzodiazepines	35 721 (16)	31 853 (16)	3 868 (26)	<0,001
Hypnotics & Sedatives	48 117 (22)	42 660 (21)	5 457 (36)	<0,001

For peer review only - http://bmjopen.bmj.com/site/about/guidelines.xhtml

Antidepressants	41 008 (19)	36 549 (18)	4 459 (30)	<0,001
Psychostimulants	1 431 (0.7)	1 270 (0.6)	161 (1.1)	<0,001
Anti-dementia drugs	2 171 (1.0)	1 867 (0.9)	304 (2.0)	<0,001
**Type of surgery – no. (%[1])**				< 0.001
Acute surgery *	64 673 (30)	59 259 (29)	5 414 (36)	
Elective surgery	152 204 (70)	142 537 (71)	9 667 (64)	
Cancer surgery	43 881 (20)	40 613 (20)	3 268 (22)	<0.001
Time of surgery – min. (IQR)	82 (50, 129)	82 (50, 128)	88 (55, 138)	<0,001
**Procedure – no (%[1])**				<0.001
Ophthalmic surgery	3 228 (1.5)	3 167 (1.6)	611 (0.4)	
Neurosurgery	14 052 (6.5)	13 019 (6.5)	1 033 (6.9)	
Endocrine surgery	7 573 (3.5)	7 429 (3.7)	144 (1.0)	
Ear, nose & throat surgery	6 839 (3.2)	6 666 (3.3)	173 (1.2)	
Oral & maxillofacial surgery	8 729 (4.0)	8 331 (4.1)	398 (2.6)	
Pulmonary surgery	2 244 (1.0)	2 023 (1.0)	221 (1.2)	
Breast surgery	12 635 (5.8)	12 104 (6.0)	531 (3.5)	
Gastrointestinal surgery	43 112 (20)	40 521 (20)	2 591 (17)	
Urologic surgery	23 642 (11)	22 509 (11)	1 133 (7.5)	
Gynaecologic surgery	18 94 (8.8)	18 412 (9.1)	582 (3.9)	
Orthopaedic surgery	61 360 (28)	54 003 (27)	7 357 (49)	
Vascular surgery	8 450 (3.9)	7 965 (4.0)	485 (3.2)	
Dermatologic surgery	6 019 (2.8)	5 647 (2.8)	372 (2.5)	
**Opioid type at discharge – no. (%[1])**				
Opiates, e.g., Morphine	57 015 (26)	48 882 (24)	8 133 (54)	<0.001
Fentanyl & Ketogan	1 179 (0.5)	922 (0.5)	257 (1.7)	<0.001
Methadone	7 675 (3.5)	6 747 (3.3)	928 (6.2)	<0.001
Buprenorphine, e.g., Suboxone	139 (0.1)	86 (0.04)	53 (0.4)	<0.001
Opioids w/ antispasmodics, e.g. Tarqiniq	317 (0.2)	269 (0.1)	48 (0.3)	<0.001
Other opioids, e.g., Tramadol	18 430 (8.5)	15 964 (7.9)	2 466 (16)	<0.001
**Year of surgery – year (%[1])**				<0.001
2007-2008	42 195 (20)	39 192 (19)	3 003 (20)	
2009-2010	46 963 (22)	43 705 (22)	3 258 (22)	
2011-2012	60 213 (28)	56 067 (28)	4 146 (27)	
2013-2014	67 506 (31)	62 832 (31)	4 674 (31)	

[1] Percentages calculated in relation to vertical study cohort, [2] Pearson's Chi-squared test, [3] Mann-Whitney U-test

† Chronic ischemic heart disease, Angina pectoris, Hypertensive disease, Cardiac arrest, Heart failure, Valve disease, Pulmonary heart disease, Cardiomyopathy, Conduction disorders/Cardiac arrhythmias, Cardiac arrest, Diseases of arteries, arterioles and capillaries.

|| Pneumonia, COPD.

‡ Acute renal failure/unspecified renal failure, Chronic renal failure, Other renal disease.

†Atherosclerosis, Aortic aneurysm and dissection, Other aneurysm, Other peripheral vascular diseases, Arterial embolism and thrombosis, Atheroembolism, Septic arterial embolism, Other disorders of arteries and arterioles, Diseases of capillaries, Disorders of arteries, arterioles and capillaries in diseases classified elsewhere.

§ Subarachnoid hemorrhage, Intracerebral hemorrhage, Other nontraumatic intracranial haemorrhage, Cerebral infarction, Acute cerebrovascular disease without cerebral infarction, Vascular occlusion without cerebral infarction, Other cerebrovascular diseases, Cerebrovascular disorders in diseases classified elsewhere, Sequelae of cerebrovascular disease.

ǂ Alzheimer's disease, Vascular dementia, Other dementia, Unspecified dementia, Non-alcoholic amnesia, Non-alcoholic delirium, mental disorders due to known physiological condition, Personality and behavioral disorders due to known physiological condition, mental disorder due to unknown somatic or organic disorder.
¶ Mental and behavioural disorders due to psychoactive substance use.
¥ Schizophrenia, Schizotypal disorder, Persistent delusional disorders, Acute and transient psychotic disorders, Induced delusional disorder, Schizoaffective disorders, Other nonorganic psychotic disorders, Unspecified nonorganic psychosis.
£ Manic episode, bipolar affective disorder, depressive episode, recurrent depressive disorder, persistent mood disorders, other affective mood disorders, unspecified affective mood disorders.
® Phobic anxiety disorders, other anxiety disorders, obsessive-compulsive disorder, reaction to severe stress and adjustment disorders, dissociative disorders, somatoform disorders, other neurotic disorders.
 Cognitive disease, substance abuse disorder, personality disorder/schizophrenia, affective disorder, anxiety disorder, Psychiatric medication within 5 years of surgery.
Ψ Collection of ≥ 1 of the listed psychiatric medications (Neuroleptics, Benzodiazepines, Hypnotics & Sedatives, Antidepressants, Antipsychotics, Anti-dementia drugs) within five years of surgery.
♦ Collection of prescription within five years of surgery.
* Acute surgery is defined as procedures that are admitted as acute surgeries. The level of acuteness can differ between right away, 6h and 72h depending on type of procedure.

For peer review only

Primary endpoint

A total number of 15,081 patients (7.0 %), opioid naïve before surgery, collected at least three opioid prescriptions during the first postoperative year; within 90 days, day 91-180 and 181-365 after surgery (Table 1).

Multivariable analysis of risk factors associated with prolonged opioid use after surgery

Estimated adjusted odds ratios (aOR) are presented in Figure 2. Odds ratios were adjusted for sex, age, comorbidities, year of surgery, and type of surgery. Postoperative prolonged opioid use was associated with female sex (aOR: 1.08. 95% CI: 1.05-1.12). In terms of the age, there was no striking trend, and the highest risk was found for patients between 50-59 years of age (aOR: 1.52. 95% CI: 1.40-1.65). Compared to a Charlson comorbidity index (CCI) of 0p, the risk increased with a higher index. CCI of 1p had an aOR of 1.31 (95% CI: 1.24-1.39) and CCI ≥ 2 had an aOR of 1.36 (95% CI: 1.30-1.43). Having a history of psychiatric disease and/or psychiatric medication use yielded an aOR of 1.94 (95% CI: 1.87-2.00). Estimated

aORs for ASA class, individual comorbidities and preoperative medications are presented in supplementary table 2.

Regarding perioperative factors acute surgery had an aOR at 1.12 (95% CI: 1.04-1.17) compared to elective surgery and cancer surgery had an aOR of 1.06 (95% CI: 1.02-1.14).

Sensitivity analysis

Several sensitivity analyses were conducted. The results remained similar when including patients that died within 12 months of surgery in the analysis. No significant time trends or regional differences were identified (supplementary table 3). As history of psychiatric disease was associated with elevated risk of prolonged opioid consumption, a restriction analysis was performed, using the adjusted model with exclusion of patients >70 years, in the upper 4th quartile (n=67 132). This yielded a higher risk estimate regarding history of psychiatric disease (aOR: 2.07. 95% CI: 1.99-2.17), indicating that the impact of a psychiatric disease on risk of prolonged opioid consumption might be higher in younger patients, detailed in supplementary table 4.

For peer review only

Discussion

In this large nation-wide retrospective perioperative cohort study, 7 % developed new prolonged opioid use. Factors associated with a higher risk of prolonged opioid use were female sex, more comorbidities, and acute surgery. Additionally, having a history of psychiatric disease was identified as the strongest risk factor.

Strengths

This is a retrospective observational cohort study investigating opioid prescription after surgery on a large Swedish population. To our knowledge, the number of included patients outnumbers many other similar studies on an international level. Utilizing the Orbit surgical planning system this study was able to include patients from different regions all around Sweden covering approximately 40% of Swedish perioperative care. Given inclusion of hospitals from different regions and levels, we believe the results to be representative of the remaining ~ 60% of Swedish care and hence, generalizable in other Swedish settings. Furthermore, the robustness and great coverage of Swedish health registers is also a strength of this study [12]. Our cohort included adults of all ages being exposed to a great variety of surgeries. Despite not having information about socioeconomic status our data should represent the actual healthcare burden of the Swedish population. The public healthcare system with services being provided according to patient need limits the selection bias that healthcare systems with various private insurances may generate.

Limitations

For peer review only

The most important weakness of this study and others in the field is the lack of consensus in defining prolonged opioid consumption. This complicates comparing results in between studies. Furthermore, there is also no consensus for how long patients should be opioid free before surgery.

Some other limitations of our study merit emphasis. First, our study was observational and therefore does not prove that our identified risk factors portray a causal relationship. Moreover, since a large part of our data was extracted from various Swedish registers misclassification bias could also have played a part in the reporting of data. However, the potential misclassification bias should be non-differential in nature and hence not push the results one direction or the other. The relatively old data is a potentially limiting factor. But, to our knowledge, there has been no major changes in Swedish postoperative pain management or legislation affecting opioid prescription patterns in recent years. This notion is also supported by the absence of differences over time in our study which makes us believe more recent data would yield similar results. Another important aspect is the fact that we are measuring dispensing of opioid prescriptions as a proxy for opioid consumption. This study does not portray patient compliance to their prescription. However, regardless of actual opioid consumption dispensing of prescriptions are problematic in and of themselves as it increases the number of opioids circulating in communities. Undeniably, we cannot account for surreptitiously opioid usage either and lack data of in-hospital drug use, something that could influence postoperative opioid usage.

Postoperative prolonged opioid use

Compared to others, this study found a higher occurrence of new prolonged opioid use after surgery [4, 6, 9, 15]. Two cohort studies of patients aged 66 or older conducted in the U.S.

For peer review only

and Canada found new postoperative prolonged opioid use at about 6% and 3%, respectively [4, 9]. Sun *et al.,* studied patients between the age of 18-64 and found prolonged opioid use at around 1% [15]. The discrepancy in occurrence of postoperative prolonged opioid use between these studies can at least partially be explained by differences in definitions of prolonged opioid use and how long a patient must be opioid naïve for. Sun *et al.,* used the most conservative definition of prolonged use where patients needed to fill 10 or more prescriptions or more than 120 days' supply of an opioid in the first year after surgery [15]. While this definition captures the patients with a high postsurgical opioid consumption it may underestimate the risk for patients with a lower, yet problematic, use. Although some patients will need opioids to manage postoperative acute pain, this period should be limited. Previous studies have shown that half of patients using opioids for longer than 90 days persist in their usage for many years after initiation [16, 17]. This highlights the importance of restrictiveness in prescribing opioids. Two of these studies only included privately insured patients [4, 15]. This introduces a bias in only selecting patients with the ability to afford a private insurance, potentially yielding a healthier population that is not generalizable to the actual population of the country. The lack of private insurances in a Swedish cohort is another strength of this study which adds additional insight to the field. Another aspect that could lead to differences in results between these studies is the lack of consensus regarding how to handle patients that died before the end of follow-up. We excluded these patients before analysis to avoid competing risk. In the Canadian cohort patients that were unable to meet the primary outcome were also excluded [9] whereas the three other studies do not state this [4, 6, 15].

Patient related risk factors for postoperative opioid use

The risk of prolonged opioid use was higher for women. This is not a consistent finding across the literature as other studies have found male sex [15] to be a greater risk factor, or no

For peer review only

significant gender difference [4, 8, 9]. For comorbidities, a higher CCI-score generated a higher aOR. We did not find a distinct trend regarding age where patients between 50-59 years of age had the highest aOR. Other studies have found both the oldest and the youngest population to be at higher risk, but also no significant difference in terms of age [4, 8, 9, 15]. History of psychiatric disease and/or medication led to almost a two-fold risk increase. Excluding the oldest population in a restriction analysis further increased the aOR for history of psychiatric disease indicating that the impact of psychiatric disease as a risk factor for new postoperative prolonged opioid use is stronger in younger patients (supplementary table 4). In line with this, other studies have found an association between preoperative benzodiazepine or antidepressant use, and prolonged postoperative opioid consumption [10, 18]. Our results, alongside aforementioned studies, indicate that clinicians must be aware of this relationship when prescribing postoperative opioids.

Surgery related risk factors for postoperative opioid use

Acute surgery had a higher occurrence of postoperative opioid use compared to elective surgery. Being exposed to cancer surgery yielded an aOR of 1.06 (95% CI: 1.02-1.14). A patient going through cancer surgery is a potential confounder in our study as treating cancer patients with opioids often is unavoidable. Partly, we tried to account for this by excluding patients prescribed opioids up to 180 days before surgery. Moreover, since individuals dying up to a year after surgery were excluded, we were hopefully able to account for unsuccessful surgical removal of the tumour and deterioration of advanced stages of malignancies. Our finding of a very low risk increase for cancer surgery speaks against this being a significant confounder.

For peer review only

Conclusion

In this large national observational cohort study, 7% developed new prolonged opioid use postoperatively. We identified several potential risk factors where a history of psychiatric disease was the most important. Data on these susceptible patients could help clinicians reduce the number of prolonged opioid users by adapting their analgesic and preventative strategies. Clinicians need be aware of the risks, have a plan for tapering, and realize that every opioid prescription has the potential of turning a non-user into a long-term user.

For peer review only

Data availability statement

Data may be obtained from a third party and are not publicly available. Data from Swedish health registers are available to all researchers upon making an application.

Ethics statements

Patient consent for publication

Not applicable.

Ethics approval

The study protocol was approved by the Regional Ethics Committee of Stockholm, Sweden (2014/1306-31/3).

Author Contributions

FL, MB and LH were responsible for the study concept and design. LH did the data collection and data cleaning. FL and LH did the data analysis. FL drafted the manuscript. MB, EL and LH did critical review of the manuscript. All the authors interpreted the data and approved the final manuscript as submitted and agree to be accountable for all aspects of the work.

Funding Statement

This research received no specific grant from any funding agency in the public, commercial or not-for-profit sectors.

For peer review only

Competing Interests Statement

None of the authors have any competing interests to declare.

For peer review only

References

[1] A. N. Nafziger and R. L. Barkin, "Opioid Therapy in Acute and Chronic Pain," *J Clin Pharmacol,* vol. 58, no. 9, pp. 1111-1122, Sep 2018, doi: 10.1002/jcph.1276.

[2] T. G. Weiser *et al.*, "Estimate of the global volume of surgery in 2012: an assessment supporting improved health outcomes," *Lancet,* vol. 385 Suppl 2, p. S11, Apr 27 2015, doi: 10.1016/s0140-6736(15)60806-6.

[3] C. E. Callinan, M. D. Neuman, K. E. Lacy, C. Gabison, and M. A. Ashburn, "The Initiation of Chronic Opioids: A Survey of Chronic Pain Patients," *J Pain,* vol. 18, no. 4, pp. 360-365, Apr 2017, doi: 10.1016/j.jpain.2016.11.001.

[4] C. M. Brummett *et al.*, "New Persistent Opioid Use After Minor and Major Surgical Procedures in US Adults," *JAMA Surg,* vol. 152, no. 6, p. e170504, Jun 21 2017, doi: 10.1001/jamasurg.2017.0504.

[5] M. J. Hartwell, R. S. Selley, M. A. Terry, and V. K. Tjong, "Can We Eliminate Opioid Medications for Postoperative Pain Control? A Prospective, Surgeon-Blinded, Randomized Controlled Trial in Knee Arthroscopic Surgery," *Am J Sports Med,* vol. 48, no. 11, pp. 2711-2717, Sep 2020, doi: 10.1177/0363546520941861.

[6] A. Alam, T. Gomes, H. Zheng, M. M. Mamdani, D. N. Juurlink, and C. M. Bell, "Long-term analgesic use after low-risk surgery: a retrospective cohort study," *Arch Intern Med,* vol. 172, no. 5, pp. 425-30, Mar 12 2012, doi: 10.1001/archinternmed.2011.1827.

[7] K. S. Ladha *et al.*, "Opioid Prescribing After Surgery in the United States, Canada, and Sweden," *JAMA Netw Open,* vol. 2, no. 9, p. e1910734, Sep 4 2019, doi: 10.1001/jamanetworkopen.2019.10734.

[8] N. M. Edwards, C. Varnum, S. Overgaard, L. Nikolajsen, C. F. Christiansen, and A. B. Pedersen, "Risk factors for new chronic opioid use after hip fracture surgery: a Danish nationwide cohort study from 2005 to 2016 using the Danish multidisciplinary hip fracture registry," *BMJ Open,* vol. 11, no. 3, p. e039238, Mar 8 2021, doi: 10.1136/bmjopen-2020-039238.

[9] H. Clarke, N. Soneji, D. T. Ko, L. Yun, and D. N. Wijeysundera, "Rates and risk factors for prolonged opioid use after major surgery: population based cohort study," *BMJ,* vol. 348, p. g1251, Feb 11 2014, doi: 10.1136/bmj.g1251.

[10] T. J. Rajamäki, T. Moilanen, P. A. Puolakka, A. Hietaharju, and E. Jämsen, "Is the Preoperative Use of Antidepressants and Benzodiazepines Associated with Opioid and Other Analgesic Use After Hip and Knee Arthroplasty?," *Clin Orthop Relat Res,* vol. 479, no. 10, pp. 2268-2280, Oct 1 2021, doi: 10.1097/corr.0000000000001793.

[11] J. F. Ludvigsson, P. Otterblad-Olausson, B. U. Pettersson, and A. Ekbom, "The Swedish personal identity number: possibilities and pitfalls in healthcare and medical research," *Eur J Epidemiol,* vol. 24, no. 11, pp. 659-67, 2009, doi: 10.1007/s10654-009-9350-y.

[12] J. F. Ludvigsson *et al.*, "External review and validation of the Swedish national inpatient register," *BMC Public Health,* vol. 11, p. 450, Jun 9 2011, doi: 10.1186/1471-2458-11-450.

[13] Socialstyrelsen. "Läkemedelsregistret." Socialstyrelsen. https://www.socialstyrelsen.se/statistik-och-data/register/lakemedelsregistret/ (accessed 2022-05-24, 2022).

[14] Socialstyrelsen. "Dödsorsaksregistret." https://www.socialstyrelsen.se/statistik-och-data/register/dodsorsaksregistret/ (accessed 2022-05-24, 2022).

[15] E. C. Sun, B. D. Darnall, L. C. Baker, and S. Mackey, "Incidence of and Risk Factors for Chronic Opioid Use Among Opioid-Naive Patients in the Postoperative Period," *Jama Internal Medicine,* vol. 176, no. 9, pp. 1286-1293, Sep 2016, doi: 10.1001/jamainternmed.2016.3298.

[16] M. Von Korff *et al.*, "De facto long-term opioid therapy for noncancer pain," *Clin J Pain,* vol. 24, no. 6, pp. 521-7, Jul-Aug 2008, doi: 10.1097/AJP.0b013e318169d03b.

[17] B. C. Martin, M. Y. Fan, M. J. Edlund, A. Devries, J. B. Braden, and M. D. Sullivan, "Long-term chronic opioid therapy discontinuation rates from the TROUP study," *J Gen Intern Med,* vol. 26, no. 12, pp. 1450-7, Dec 2011, doi: 10.1007/s11606-011-1771-0.

For peer review only - http://bmjopen.bmj.com/site/about/guidelines.xhtml

[18] C. A. Rishel, Y. Zhang, and E. C. Sun, "Association Between Preoperative Benzodiazepine Use and Postoperative Opioid Use and Health Care Costs," *JAMA Netw Open,* vol. 3, no. 10, p. e2018761, Oct 1 2020, doi: 10.1001/jamanetworkopen.2020.18761.

For peer review only

Figure legends

Figure 1. Participant flowchart

Figure 1. Risk factors for developing prolonged opioid use after surgery among patients with no opioid use 180 days before surgery. Odds ratios adjusted for sex, age, comorbidities (charlson comorbidity index) and surgical procedure.

** A diagnosis of ≥ 1 of the psychiatric diseases listed (Table 1) and/or collected a prescription of ≥ of the listed psychiatric medications (Table 1) within five years of surgery.

For peer review only

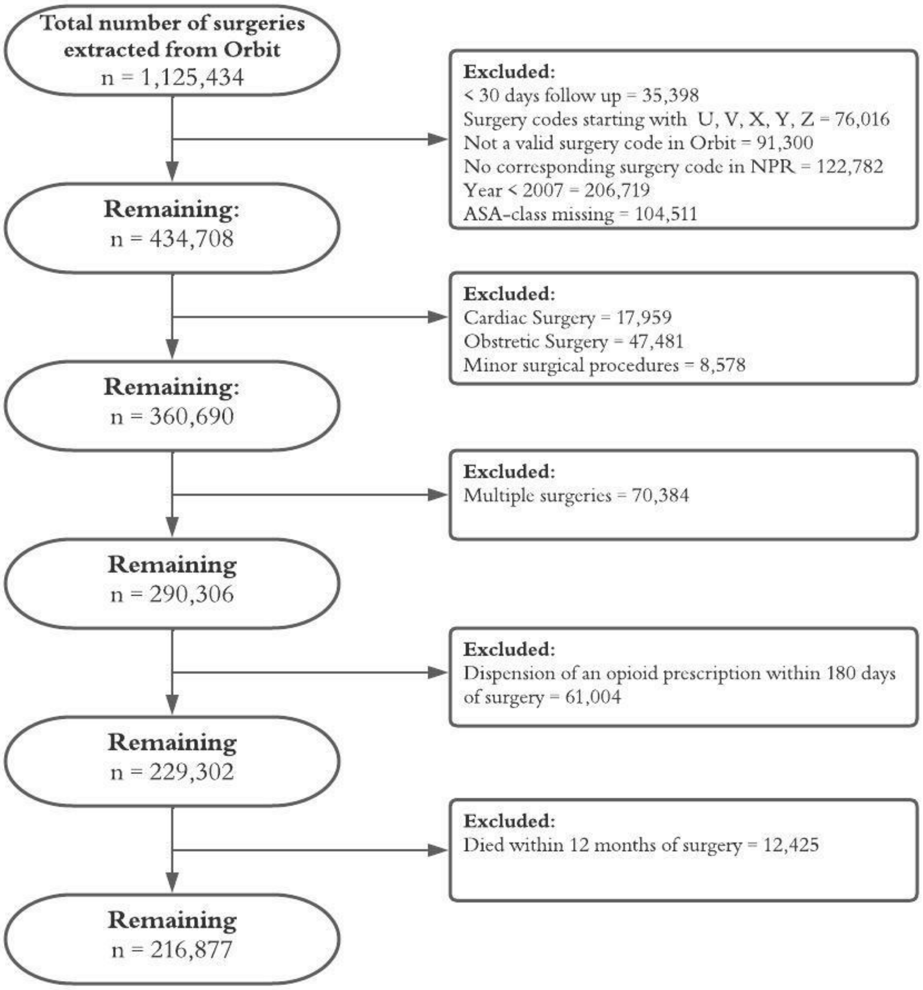

Figure 1. Participant flowchart

142x152mm (160 x 160 DPI)

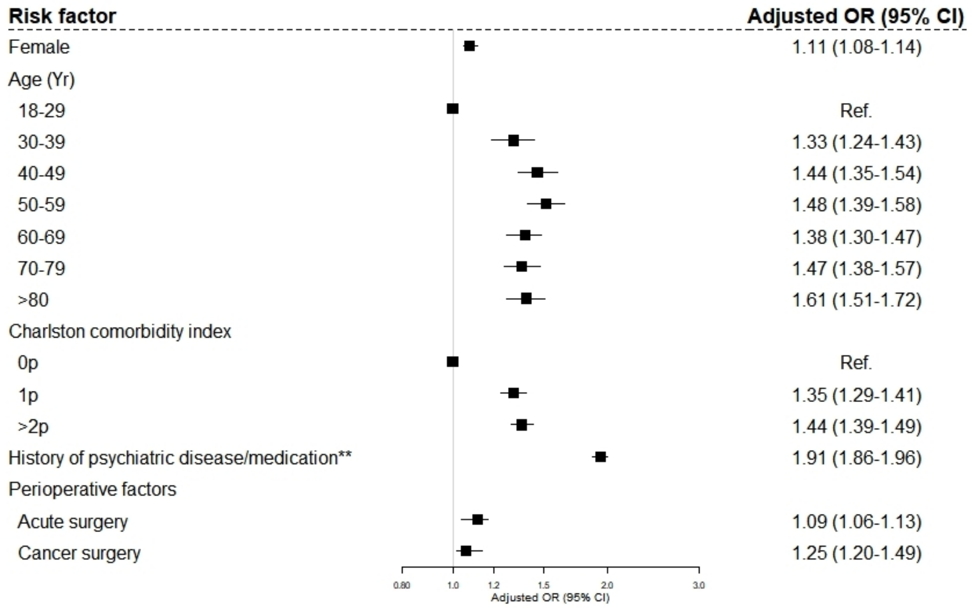

Figure 2. Risk factors for developing prolonged opioid use after surgery among patients with no opioid use 180 days before surgery. Odds ratios adjusted for sex, age, comorbidities (charlson comorbidity index) and surgical procedure.
** A diagnosis of ≥ 1 of the psychiatric diseases listed (Table 1) and/or collected a prescription of ≥ of the listed psychiatric medications (Table 1) within five years of surgery.

228x158mm (96 x 96 DPI)

Supplementary table 1: Included and excluded surgery codes.

Included surgery codes:

A – nervous system
B – endocrine system
C – eye and adjacent structures
D - ear, nose, and throat
G - pulmonary
H – mammary gland
J - gastrointestinal
K - urology
L - gynecology
N - musculoskeletal system
P - vascular
Q – dermatology

Excluded surgery codes:

F – cardiac
M – obstetric
T – minor surgical procedures
U – transluminal endoscopy
V – Surgery on aorta, peripheral vessels, and lymphatic system.
X – diagnostic procedures associated with surgery
Y – removal of organs for transplant
Z – additional codes

Minor surgical procedures include:

Neurological procedures: lumbar puncture, peripheral nerve block, sympathetic block TAW and other minor neurosurgical procedure.

Endocrine surgery: puncture of thyroid gland, needle biopsy of thyroid gland, aspiration cytology of thyroid gland, puncture of parathyroid gland, needle biopsy of parathyroid gland, needle biopsy of adrenal gland, aspiration cytology of adrenal gland, other minor procedure in endocrine surgery

Eye surgery: needle biopsy of orbit, aspiration cytology of orbit, retrobulbar therapeutic injection into orbit, peribulbar therapeutic injection into orbit, needle biopsy of eyelid, simple epilation of cilia, therapeutic injection into eyelid, removal of ptosis suture from eyelid, adjustment of ptosis suture, needle biopsy of lacrimal gland, probing of lacrimal canaliculus or of nasolacrimal duct, intubation of lacrimal canaliculus or intubation of nasolacrimal duct, therapeutic injection into extraocular muscle, subconjunctival therapeutic injection, protection of cornea and sclera with soft lens, removal of sutures from cornea, laser suture lysis in cornea or sclera, needle aspiration of aqueous from anterior chamber, needle biopsy of iris, needle biopsy of ciliary body, needle biopsy of choroid, needle biopsy of vitreous body, needle biopsy of retina, other minor procedure in eye surgery

Ear, nose and throat: biopsy of auricle or external auditory canal, needle biopsy of auricle or external auditory canal, removal of foreign body from external auditory canal, needle biopsy of tympanic membrane, needle biopsy of nose, removal of foreign body from cavity of nose, needle biopsy of septum of nose, needle biopsy of maxillary antrum, puncture and irrigation of maxillary antrum, needle biopsy of ethmoidal bone and sinus, needle biopsy of frontal sinus, needle biopsy of sphenoidal sinus, needle biopsy of larynx, other minor procedure in surgery of ear, nose and larynx

For peer review only

Breast surgery: needle biopsy of mamilla, needle biopsy of mammary gland, other minor procedure in surgery of mammary gland

Gastroenterological surgery: puncture of peritoneum, laparocentesis, percutaneous peritoneal irrigation, percutaneous introduction of peritoneal dialysis catheter, removal of peritoneal dialysis catheter, percutaneous local drainage of peritoneal cavity, introduction of intraperitoneal injection port, removal of intraperitoneal injection port, insertion of ballon tube for tamponade of oesophageal varices, insertion of nasogastric or naso-gastroduodenal tube, insertion of other tube into stomach or duodenum, change of gastrostomy tube, insertion of jejunal tube, insertion of jejunal tube through gastrostomy, peroral biopsy of small intestine, reduction of intestinal intussusception using barium enema, digital extraction of impacted faeces, removal of foreign body from rectum, percutaneous needle biopsy of liver, percutaneous destruction of lesion of liver, intraoperative cholangiography, laparoscopic cholangiography, percutaneous needle biopsy of pancreas, percutaneous drainage of pseudocyst or abscess of pancreas, needle biopsy of spleen, other minor procedure in gastroenterological surgery.

Urology: needle biopsy of kidney or pelvis of kidney, aspiration cytology of kidney, percutaneous puncture of kidney or pelvis of kidney, needle biopsy of bladder, percutaneous puncture of bladder, catheterisation of bladder, dilatation of urethra, needle biopsy of prostate, aspiration cytology of prostate, puncture of prostate, needle biopsy of testis, aspiration cytology of testis, puncture of testis, needle biopsy of epididymis, aspiration cytology of epididymis, puncture of epididymis, puncture of scrotum, other minor surgical procedure in urology

Gynecology: needle biopsy of ovary, needle biopsy of Fallopian tube, insertion of intrauterine contraceptive device, dilatation of vagina, removal of foreign body from vagina in childhood, transvaginal puncture of cul-de-sac, other minor surgical procedure in gynaecology

Orthopedic surgery: puncture or needle biopsy of soft tissue, simple incision of soft tissue, arthrocentesis, injection of diagnostic or therapeutic substance into joint, aspiration of bone marrow, drill biopsy of bone marrow, soft bandage, prefabricated orthosis, custom moulded splint, custom moulded cast, major custom moulded cast, other orthopedic bandage, external traction, implantation of skeletal marker

Peripheral vessels and lymphatic system: phlebotomy, open cannulation of peripheral vein, sclerotherapy of varicose veins, insertion of central venous catheter through external or internal jugular vein, insertion of central venous catheter through subclavian or brachiocephalic vein, biopsy of superficial lymph node, needle biopsy of lymph node, other minor surgical procedure on peripheral vessel or lymphatic system, biopsy of artery or vein, implantation of vascular injection port, removal of vascular injection port

Dermatology: other minor procedure on skin, biopsy of skin, needle biopsy of skin, removal of sutures of skin, adjustment of volume of tissue expander

For peer review only

Supplementary table 2. Risk factors for developing prolonged opioid use after surgery among patients with no opioid use 180 days before surgery. Odds ratios adjusted for sex, age and surgical procedure.

**Risk factor**	**Crude OR (95% CI)**	**Adjusted OR (95% CI)**[1]
**Patient factors**		
Female	1.08 (1.05-1.12)	1.16 (1.12-1.21)
Age (yr) 18-29	Ref.	Ref.
30-39	1.31 (1.19-1.44)	1.43 (1.30-1.57)
40-49	1.52 (1.40.-1.66)	1.67 (1.53-1.82)
50-59	1.90 (1.75-2.06)	1.91 (1.76-2.07)
60-69	1.96 (1.82-2.11)	1.85 (1.71-2.00)
70-79	2.19 (2.03-2.36)	1.92 (1.77-2.07)
> 80	2.80 (2.59-3.03)	2.16 (1.99-2.34)
**Preoperative data**		
Charlson comorbidity index 0p	Ref.	Ref.
1p	1.65 (1.56-1.74)	1.43 (1.35-1.51)
≥ 2p	1.42 (1.37-1.47)	1.69 (1.63-1.76)
ASA classification 1	Ref.	Ref.
2	1.83 (1.76-1.92)	1.65 (1.57-1.73)
3	2.57 (2.45-2.69)	2.25 (2.13-2.38)
4	3.07 (2.71-3.47)	2.76 (2.43-3.14)
Comorbidities		
Heart disease[†]	1.54 (1.48-1.59)	1.32 (1.27-1.37)
Lung disease[||]	1.79 (1.69-1.91)	1.59 (1.49-1.70)
Renal disease[‡]	1.35 (1.24-1.48)	1.46 (1.33-1.60)
Diabetes mellitus	1.53 (1.46-1.61)	1.40 (1.33-1.47)
Vascular disease[↑]	1.44 (1.32-1.56)	1.44 (1.32-1.57)
Cerebrovascular disease[§]	1.50 (1.40-1.62)	1.25 (1.16-1.35)
History of psychiatric disease		
Substance abuse[¶]	1.97 (1.83-2.13)	1.99 (1.84-2.15)
Cognitive disease[ǂ]	2.15 (1.96-2.36)	1.50 (1.36-1.65)
Personality disorder, Schizophrenia[¥]	1.23 (1.12-1.35)	1.52 (1.38-1.67)
Affective disorder[£]	1.63 (1.52-1.75)	1.70 (1.59-1.83)
Anxiety disorder[®]	1.58 (1.47-1.69)	1.81 (1.68-1.95)
Preoperative Medication[♦]		
Neuroleptics	1.75 (1.63-1.89)	1.58 (1.46-1.71)
Benzodiazepines	1.84 (1.77-1.91)	1.75 (1.68-1.82)
Hypnotics & Sedatives	2.11 (2.04-2.19)	1.95 (1.89-2.02)
Antidepressants	1.90 (1.83-1.97)	1.80 (1.73-1.86)
Psychostimulants	1.70 (1.44-2.01)	2.18 (1.84-2.58)
History of psychiatric disease and/or medication**	2.10 (2.03-2.17)	1.98 (1.92-2.05)
**Perioperative factors**		
Acute surgery*	1.35 (1.30-1.39)	1.04 (1.01-1.09)
Cancer surgery	1.10 (1.05-1.14)	1.08 (1.04-1.14)

For peer review only - http://bmjopen.bmj.com/site/about/guidelines.xhtml

† Chronic ischemic heart disease, Angina pectoris, Hypertensive disease, Cardiac arrest, Heart failure, Valve disease, Pulmonary heart disease, Cardiomyopathy, Conduction disorders/Cardiac arrhythmias, Cardiac arrest, Diseases of arteries, arterioles and capillaries.
|| Pneumonia, COPD.
‡ Acute renal failure/unspecified renal failure, Chronic renal failure, Other renal disease.
↑Atherosclerosis, Aortic aneurysm and dissection, Other aneurysm, Other peripheral vascular diseases, Arterial embolism and thrombosis, Atheroembolism, Septic arterial embolism, Other disorders of arteries and arterioles, Diseases of capillaries, Disorders of arteries, arterioles and capillaries in diseases classified elsewhere.
§ Subarachnoid hemorrhage, Intracerebral hemorrhage, Other nontraumatic intracranial haemorrhage, Cerebral infarction, Acute cerebrovascular disease without cerebral infarction, Vascular occlusion without cerebral infarction, Other cerebrovascular diseases, Cerebrovascular disorders in diseases classified elsewhere, Sequelae of cerebrovascular disease.
¶ Mental and behavioural disorders due to psychoactive substance use.
ƚ Alzheimer's disease, Vascular dementia, Other dementia, Unspecified dementia, Non-alcoholic amnesia, Non-alcoholic delirium, mental disorders due to known physiological condition, Personality and behavioral disorders due to known physiological condition, mental disorder due to unknown somatic or organic disorder.
¥ Schizophrenia, Schizotypal disorder, Persistent delusional disorders, Acute and transient psychotic disorders, Induced delusional disorder, Schizoaffective disorders, Other nonorganic psychotic disorders, Unspecified nonorganic psychosis.
£ Manic episode, bipolar affective disorder, depressive episode, recurrent depressive disorder, persistent mood disorders, other affective mood disorders, unspecified affective mood disorders.
® Phobic anxiety disorders, other anxiety disorders, obsessive-compulsive disorder, reaction to severe stress and adjustment disorders, dissociative disorders, somatoform disorders, other neurotic disorders.
Ⱶ Cognitive disease, substance abuse disorder, personality disorder/schizophrenia, affective disorder, anxiety disorder, Psychiatric medication within 5 years of surgery..
♦ Collection of prescription within five years of surgery.
* Acute surgery is defined as procedures that are admitted as acute surgeries. The level of acuteness can differ between right away, 6h and 72h depending on type of procedure.
** A diagnosis of ≥ 1 of the psychiatric diseases listed (Table 1) and/or collected a prescription of ≥ of the listed psychiatric medications (Table 1) within five years of surgery.

For peer review only

Supplementary table 3. *Baseline characteristics of surgical cohort at inclusion (n=216,877) and proportion of new prolonged opioid consumption.*

		New Prolonged Opioid Consumption		
	Total n = 216 877 (%)	No n = 201 796 (93)	Yes n = 15 081 (7.0)	*p*-value[2,3]
**Procedure – no (%[1])**				<0.001
Ophthalmic surgery	3 228	3 167 (98)	611 (1.9)	
Neurosurgery	14 052	13 019 (93)	1 033 (7.4)	
Endocrine surgery	7 573	7 429 (98)	144 (1.9)	
Ear, nose & throat surgery	6 839	6 666 (97)	173 2.5)	
Oral & maxillofacial surgery	8 729	8 331 (95)	398 (4.6)	
Pulmonary surgery	2 244	2 023 (90)	221 (9.9)	
Breast surgery	12 635	12 104 (96)	531 (3.5)	
Gastrointestinal surgery	43 112	40 521 (94)	2 591 (6)	
Urologic surgery	23 642	22 509 (95)	1 133 (4.8)	
Gynecologic surgery	18 994	18 412 (97)	582 (3.1)	
Orthopedic surgery	61 360	54 003 (88)	7 357 (12)	
Vascular surgery	8 450	7 965 (94)	485 (5.7)	
Dermatologic surgery	6 019	5 647 (94)	372 (6.2)	
**Year of surgery – year (%[1])**				0.52
2007-2008	42 195	39 192 (93)	5 239 (7.1)	
2009-2010	46 963	43 705 (93)	5 641 (6.9)	
2011-2012	60 213	56 067 (93)	7 174 (6.9)	
2013-2014	67 506	62 832 (93)	7 943 (6.9)	
**Region of surgery – no (%[1])**				0.009
Stockholm & Södermanland	61 142	56 935 (93)	4 207 6.9)	
Skåne	80 964	75 147 (93)	5 817 (7.2)	
Västra Götaland	37 090	34 566 (93)	2 524 (6.8)	
Västernorrland & Västerbotten	37 681	35 148 (93)	2 533 (6.7)	

[1] Percentages calculated in relation to horizontal study cohort, [2] Pearson's Chi-squared test, [3] Mann-Whitney U-test

Supplementary table 4. Sensitivity analyses of the association between history of psychiatric disease and prolonged opioid use. Restriction of the cohort; excluding patients >70 years old (75th percentile).

Risk factor	OR* (95% CI)
History of psychiatric disease	2.07 (1.99 to 2.17)

* Adjusted for sex, age, comorbidities (charlson comorbidity index) and surgical procedure.

For peer review only

STROBE Statement—Checklist of items that should be included in reports of ***cohort studies***

	Item No	Recommendation	Page No
**Title and abstract**	1	(*a*) Indicate the study's design with a commonly used term in the title or the abstract	1
		(*b*) Provide in the abstract an informative and balanced summary of what was done and what was found	1
**Introduction**			
Background/rationale	2	Explain the scientific background and rationale for the investigation being reported	3
Objectives	3	State specific objectives, including any prespecified hypotheses	3-4
**Methods**			
Study design	4	Present key elements of study design early in the paper	5-6
Setting	5	Describe the setting, locations, and relevant dates, including periods of recruitment, exposure, follow-up, and data collection	5
Participants	6	(*a*) Give the eligibility criteria, and the sources and methods of selection of participants. Describe methods of follow-up	5
		(*b*) For matched studies, give matching criteria and number of exposed and unexposed	N/A
Variables	7	Clearly define all outcomes, exposures, predictors, potential confounders, and effect modifiers. Give diagnostic criteria, if applicable	6-7
Data sources/ measurement	8*	For each variable of interest, give sources of data and details of methods of assessment (measurement). Describe comparability of assessment methods if there is more than one group	5-6
Bias	9	Describe any efforts to address potential sources of bias	8
Study size	10	Explain how the study size was arrived at	Figure 1
Quantitative variables	11	Explain how quantitative variables were handled in the analyses. If applicable, describe which groupings were chosen and why	7
Statistical methods	12	(*a*) Describe all statistical methods, including those used to control for confounding	7
		(*b*) Describe any methods used to examine subgroups and interactions	7
		(*c*) Explain how missing data were addressed	7
		(*d*) If applicable, explain how loss to follow-up was addressed	N/A
		(*e*) Describe any sensitivity analyses	10
**Results**			
Participants	13*	(a) Report numbers of individuals at each stage of study—eg numbers potentially eligible, examined for eligibility, confirmed eligible, included in the study, completing follow-up, and analysed	Figure 1
		(b) Give reasons for non-participation at each stage	5 & Figure 1
		(c) Consider use of a flow diagram	Figure 1
Descriptive data	14*	(a) Give characteristics of study participants (eg demographic, clinical, social) and information on exposures and potential confounders	5, 9 & Table 1
		(b) Indicate number of participants with missing data for each variable of interest	Table 1

For peer review only - http://bmjopen.bmj.com/site/about/guidelines.xhtml

		(c) Summarise follow-up time (eg, average and total amount)	6
Outcome data	15*	Report numbers of outcome events or summary measures over time	9 & Table 1
Main results	16	(*a*) Give unadjusted estimates and, if applicable, confounder-adjusted estimates and their precision (eg, 95% confidence interval). Make clear which confounders were adjusted for and why they were included	9, 10 & Table 1
		(*b*) Report category boundaries when continuous variables were categorized	7-8
		(*c*) If relevant, consider translating estimates of relative risk into absolute risk for a meaningful time period	
Other analyses	17	Report other analyses done—eg analyses of subgroups and interactions, and sensitivity analyses	10
**Discussion**			
Key results	18	Summarise key results with reference to study objectives	9, 10
Limitations	19	Discuss limitations of the study, taking into account sources of potential bias or imprecision. Discuss both direction and magnitude of any potential bias	14, 15
Interpretation	20	Give a cautious overall interpretation of results considering objectives, limitations, multiplicity of analyses, results from similar studies, and other relevant evidence	11-13
Generalisability	21	Discuss the generalisability (external validity) of the study results	13-14
**Other information**			
Funding	22	Give the source of funding and the role of the funders for the present study and, if applicable, for the original study on which the present article is based	16

*Give information separately for exposed and unexposed groups.

Note: An Explanation and Elaboration article discusses each checklist item and gives methodological background and published examples of transparent reporting. The STROBE checklist is best used in conjunction with this article (freely available on the Web sites of PLoS Medicine at http://www.plosmedicine.org/, Annals of Internal Medicine at http://www.annals.org/, and Epidemiology at http://www.epidem.com/). Information on the STROBE Initiative is available at http://www.strobe-statement.org.

For peer review only - http://bmjopen.bmj.com/site/about/guidelines.xhtml
